# Supplementary material for: Estimating the epidemic reproduction number from temporally aggregated incidence data: A statistical modelling approach and software tool
Source: PLoS Comput Biol. 2023 Aug 28;19(8):e1011439. doi: 10.1371/journal.pcbi.1011439 (PMC10491397; doi:10.1371/journal.pcbi.1011439)
Supplement: S1 Appendix — (PDF) [file pcbi.1011439.s001.pdf]

Supplementary Information:
Estimating the epidemic reproduction number from temporally aggregated incidence data

Rebecca K Nash[1] MSc, Samir Bhatt[1,2] PhD, Anne Cori[1] PhD*, Pierre Nouvellet[1,3] PhD*
*Contributed equally
[1]*MRC Centre for Global Infectious Disease Analysis, Jameel Institute, School of Public Health, Imperial College London*
[2]*Section of Epidemiology, Department of Public Health, University of Copenhagen*
[2]*School of Life Sciences, University of Sussex*

Table of Contents

1. Overview 1
2. Additional results 1
   a. Influenza 2
   b. COVID-19 cases 3
   c. COVID-19 deaths 6
   d. Reported incidence patterns by weekday 7
   e. Classification tables 8
   f. Computational time 10
3. Simulation study 10
   a. Constant R_t 10
   b. Discontinuities in the reconstructed incidence data 11
   c. Time varying R_t: sudden change 12
   d. Time-varying R_t: gradual change 15
   e. Influence of R_t plotting time relative to the time window used for estimation 18
   f. Weekend effects 20
   g. Mid-aggregation variations in transmissibility 21
   h. Number of iterations 22
   i. Different temporal aggregations 23
4. Alternative approach 25
5. Zika virus disease 28
6. Alternative analysis pipelines 29

1. Overview

In this supplementary information we present further results from our analyses of influenza-like illness and COVID-19 data. We also present analyses from a simulation study, which was used to validate our method using a variety of simulated epidemic scenarios, where R_t either remained constant or varied over time. We explored the impact of weekend effects on estimates of R_t, the ability to detect genuine mid-aggregation variations in transmissibility, the application of the method to alternative temporal aggregations of data e.g., 3-day, 10-day, or two-weekly aggregations, and we discuss the number of iterations generally required to reach convergence when reconstructing daily incidence data using our Expectation-Maximisation (EM) algorithm. We also compare our method to an alternative LOESS smoothing approach, and present another real-world example using incidence data for Zika, as an example of a disease with non-respiratory transmission.

2. Additional results

As described in the main text, reported incidence data for cases of influenza-like illness, COVID-19 cases, and COVID-19 deaths was used to estimate R_t using the original EpiEstim R package[1] as well as our extended method. The reported daily data were artificially aggregated to a weekly timescale to replicate a typical scenario where data are reported on a weekly basis. Both the reported daily data and the reconstructed daily data, which was obtained from the weekly aggregations of incidence using our EM algorithm, were used to estimate R_t over daily and weekly sliding windows ending on day t.

a. Influenza

In the influenza case study, both the daily and weekly sliding R_t estimates are smoother when estimated from the reconstructed data as opposed to the reported data (Figure S1). The prominent weekly oscillations in the daily R_t estimates from the reported data are likely due to the impact of day-to-day variations in reporting, such as weekend effects, which in this dataset may be caused by factors including the military clinic's opening hours or the military personnel's working hours.[2] These intra-weekly fluctuations are lost once the data are aggregated; therefore, the reconstructed incidence and the R_t estimates based on it will be smoother and less affected by this variability (Figure S1 & Figure S2A-B).

Weekly sliding R_t estimates account for some of this variation, leading to smoother R_t estimates from both the reported and reconstructed data (Figure S1B). It is much clearer here that the estimates follow the same general trend, but once again, the R_t estimates are slightly smoother from the reconstructed data.

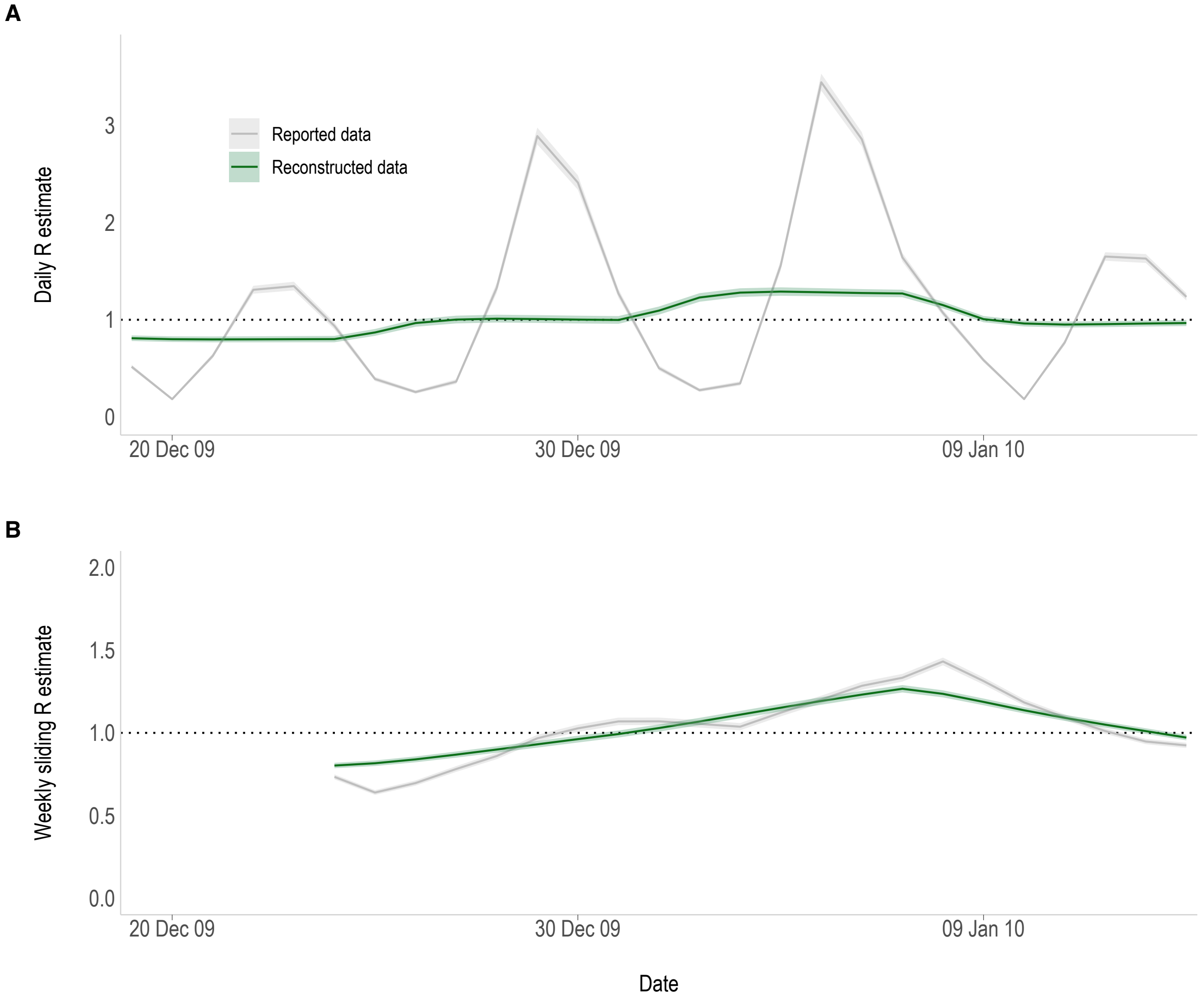

Figure S1. The A) daily and B) weekly sliding R_t estimates for influenza based on the reported (grey) and the reconstructed (green) daily data by date of presentation at the military clinic. R_t estimates start on the first day of the second aggregation window (day 8 – 18th December 2009) and are plotted at the end of the time window. Shading corresponds to the 95% credible interval of the estimates.

Bias in the R_t estimates was assessed by computing the absolute difference in the estimates from the reported and reconstructed data (Figure S2C-D). The greater differences between the R_t estimates correspond to the largest disparities between the reported and reconstructed incidence data (Figure S2).

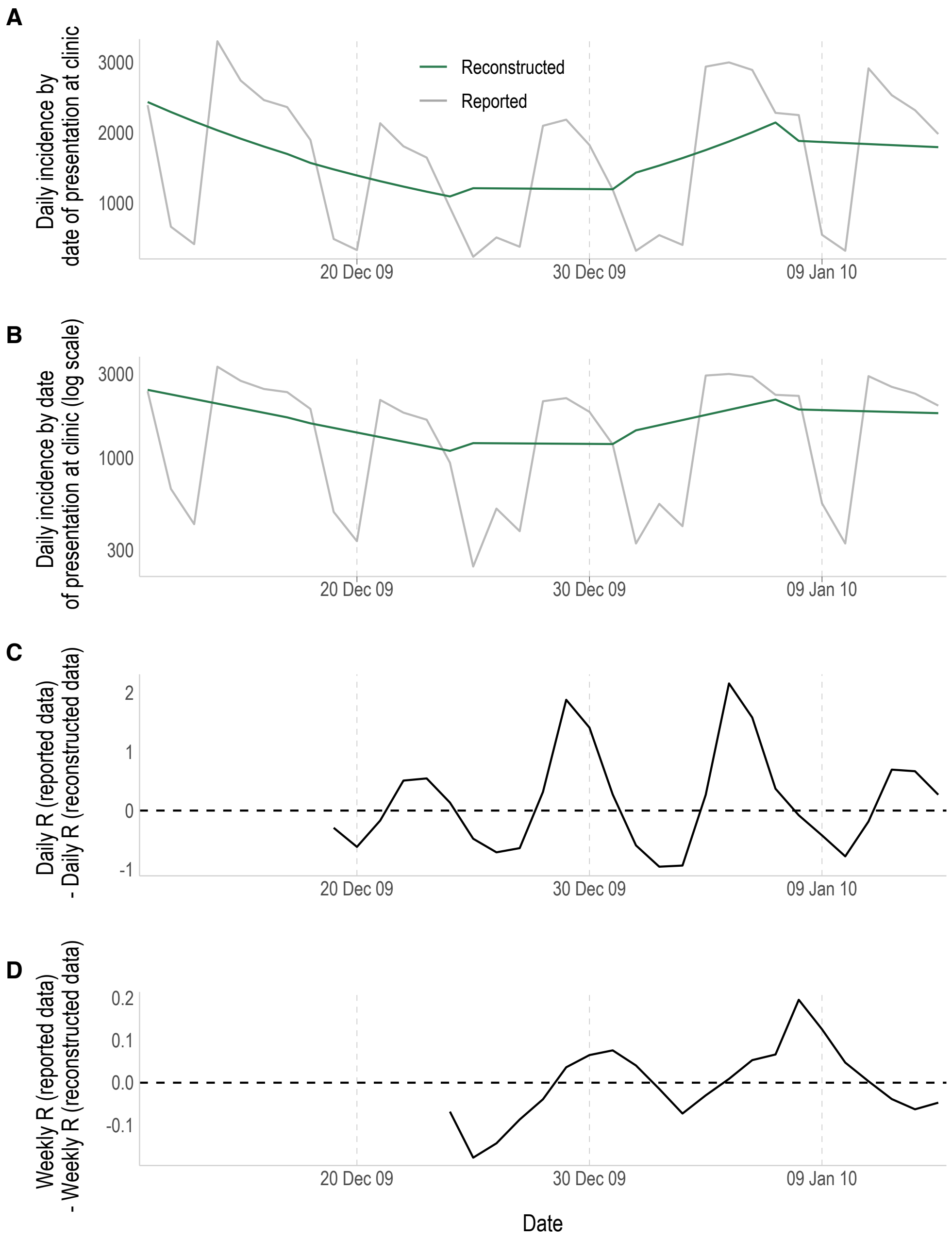

Figure S2. A-B) The reported (grey) and reconstructed (green) daily incidence of influenza by the date of presentation at the military clinic, either on a natural scale (A) or log scale (B). C-D) The absolute difference in the C) daily and D) weekly sliding R_t estimates made using the reported daily data and the reconstructed daily data. Note that the y-axis scale is different in panels C and D.

b. COVID-19 cases

In the first case study for COVID-19, the overall trends in R_t over time using the reported and reconstructed daily data were similar for both the daily and weekly sliding R_t estimates (Figure S3). The daily R_t estimates using the reconstructed data considerably smoothed out the impact of weekend effects, which were prominent in the reported incidence (Figure S3A, Figure S4A-B). The weekly sliding R_t estimates are very similar using both datasets, with some minor discrepancies e.g., around early September and mid-December 2020 (Figure S3B).

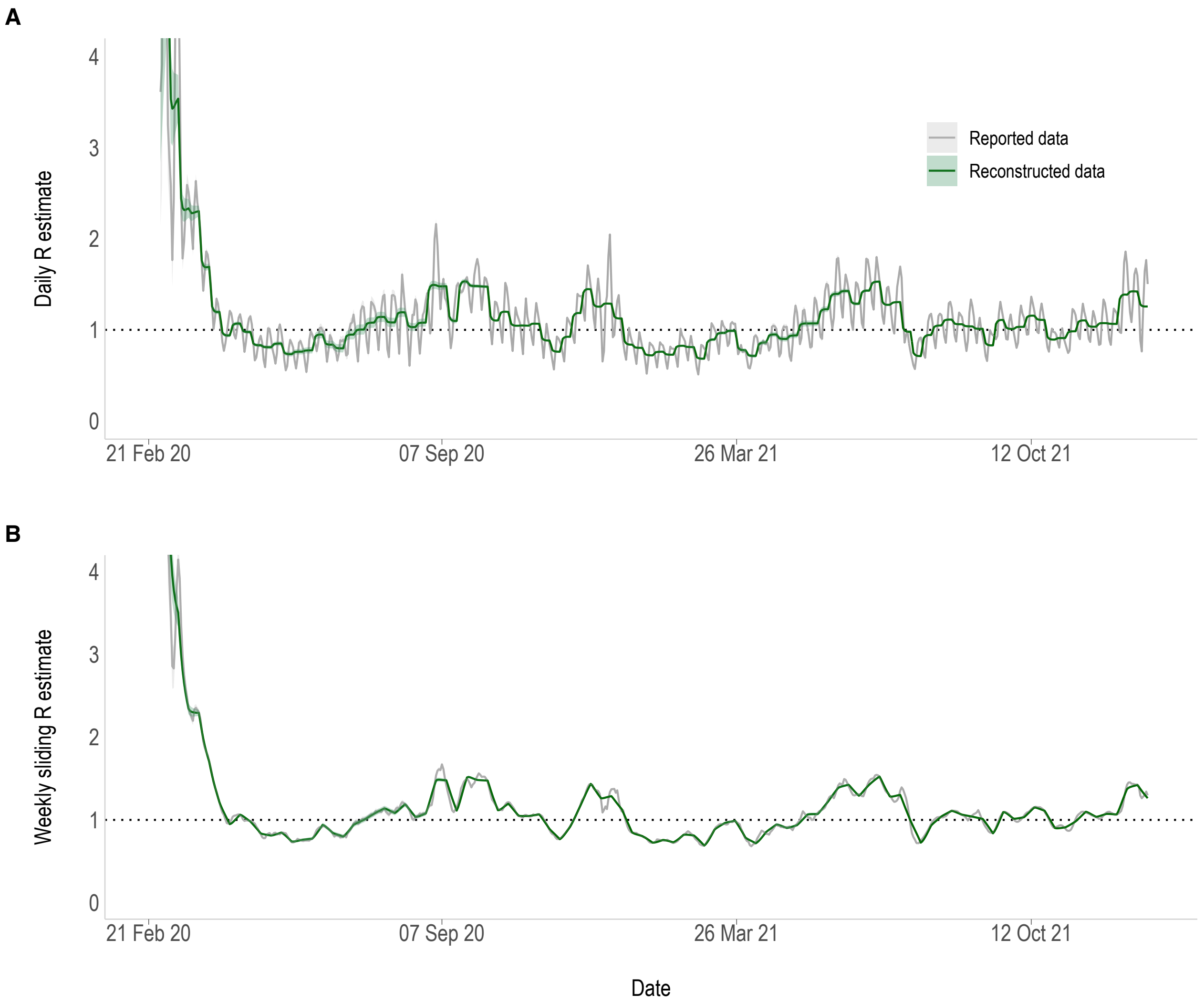

Figure S3. The A) daily and B) weekly sliding R_t estimates for COVID-19 cases based on the reported (grey) and the reconstructed (green) daily data by date of specimen. R_t estimates start on the first day of the second aggregation window (day 8 – 28th February 2020) and are plotted at the end of the time window. Shading corresponds to the 95% credible interval of the estimates. The y-axis has been cropped to a maximum of 4 for clarity.

When comparing the absolute difference in the R_t estimates made using the reported and reconstructed data, the larger spikes coincide with periods of either low incidence or more prominent weekend effects in the reported data (Figure S4).

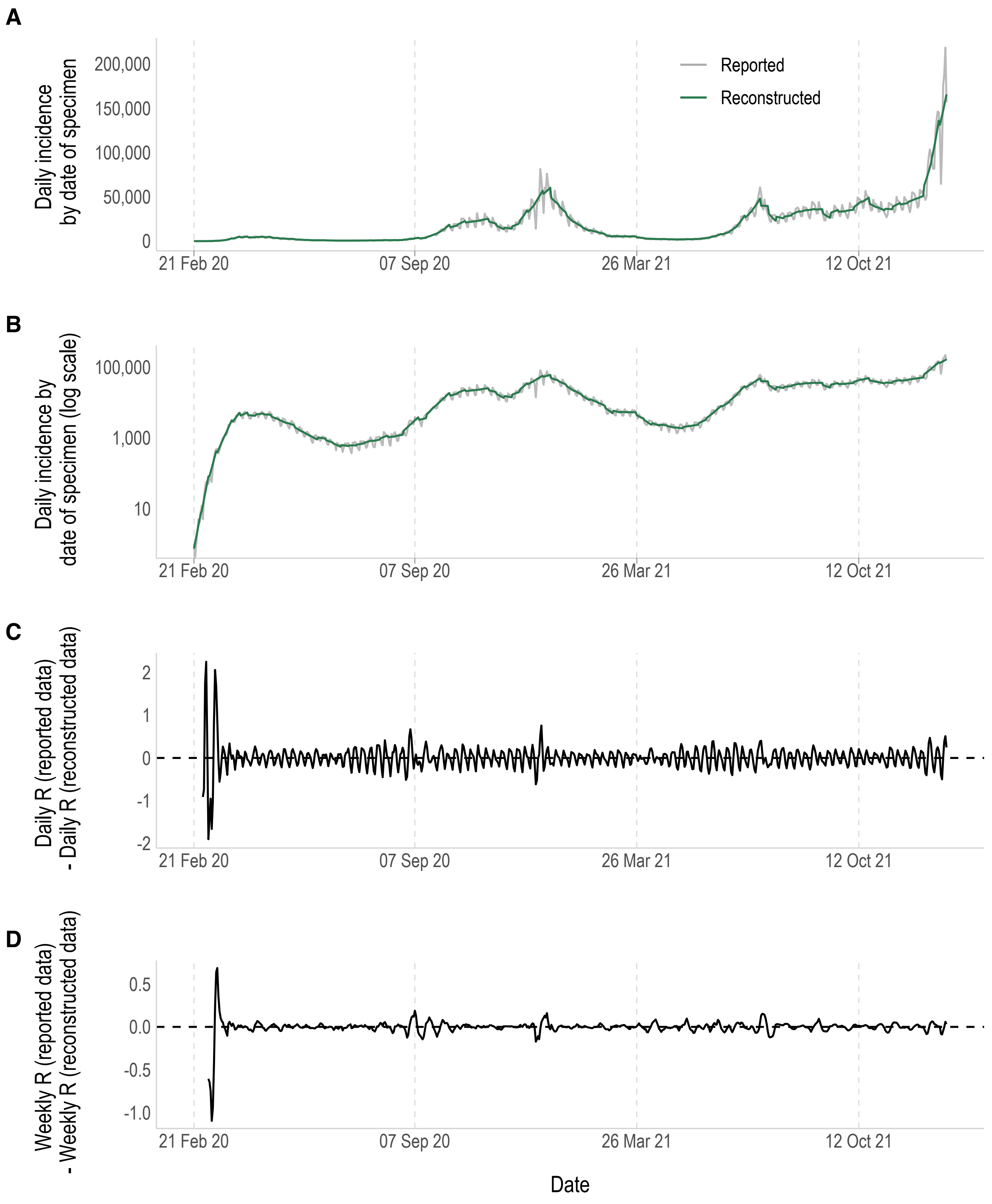

Figure S4. A-B) The reported (grey) and reconstructed (green) daily incidence of COVID-19 by the date of specimen, either on a natural scale (A) or log scale (B). C-D) The absolute difference in the C) daily and D) weekly sliding R_t estimates made using the reported data and the reconstructed data. Note that the y-axis scale is different in panels C and D.

c. COVID-19 deaths

In the second case study for COVID-19, due to the lower incidence of COVID-19 deaths compared to COVID-19 cases, there is greater uncertainty in the R_t estimates (Figure S5). There is still some indication of noise in this dataset, but the pattern appears less pronounced and more irregular than the variation caused by weekend effects as seen in the COVID-19 cases data. Unlike the data for flu and COVID-19 cases, further analysis showed that there was no pattern in reporting depending on the day of the week (Figure S7).

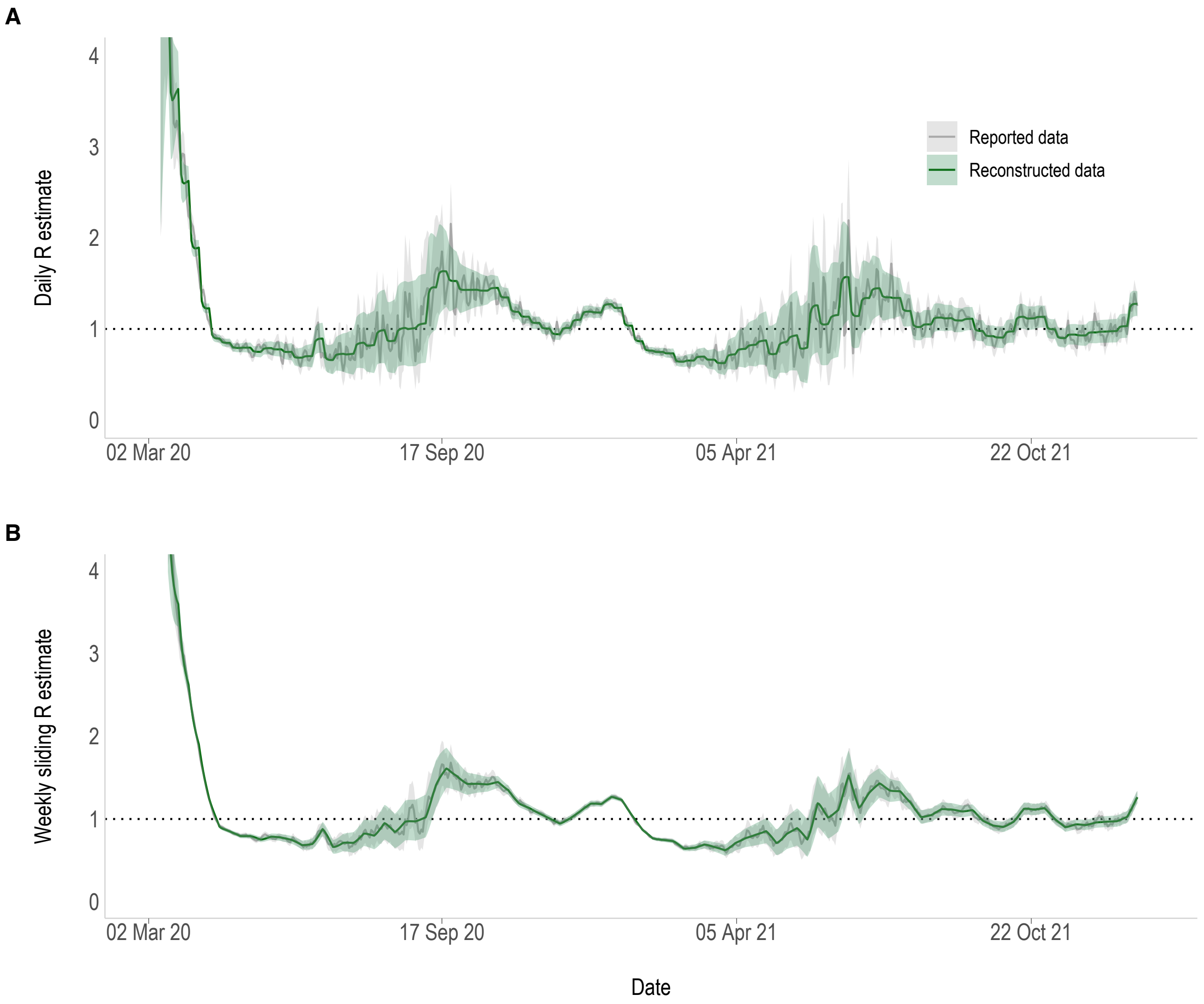

Figure S5. The A) daily and B) weekly sliding R_t estimates for COVID-19 deaths based on the reported daily data (grey) and the reconstructed daily data (green) by date of death within 28 days of a positive test. R_t estimates start on the first day of the second aggregation window (day 8 – 9th March 2020) and are plotted at the end of the time window. Shading corresponds to the 95% credible interval of the estimates. The y-axis has been cropped to a maximum of 4 for clarity.

In contrast with COVID-19 case data, the greatest differences in R_t estimates obtained from death data appear to coincide solely with periods of low incidence (Figure S6).

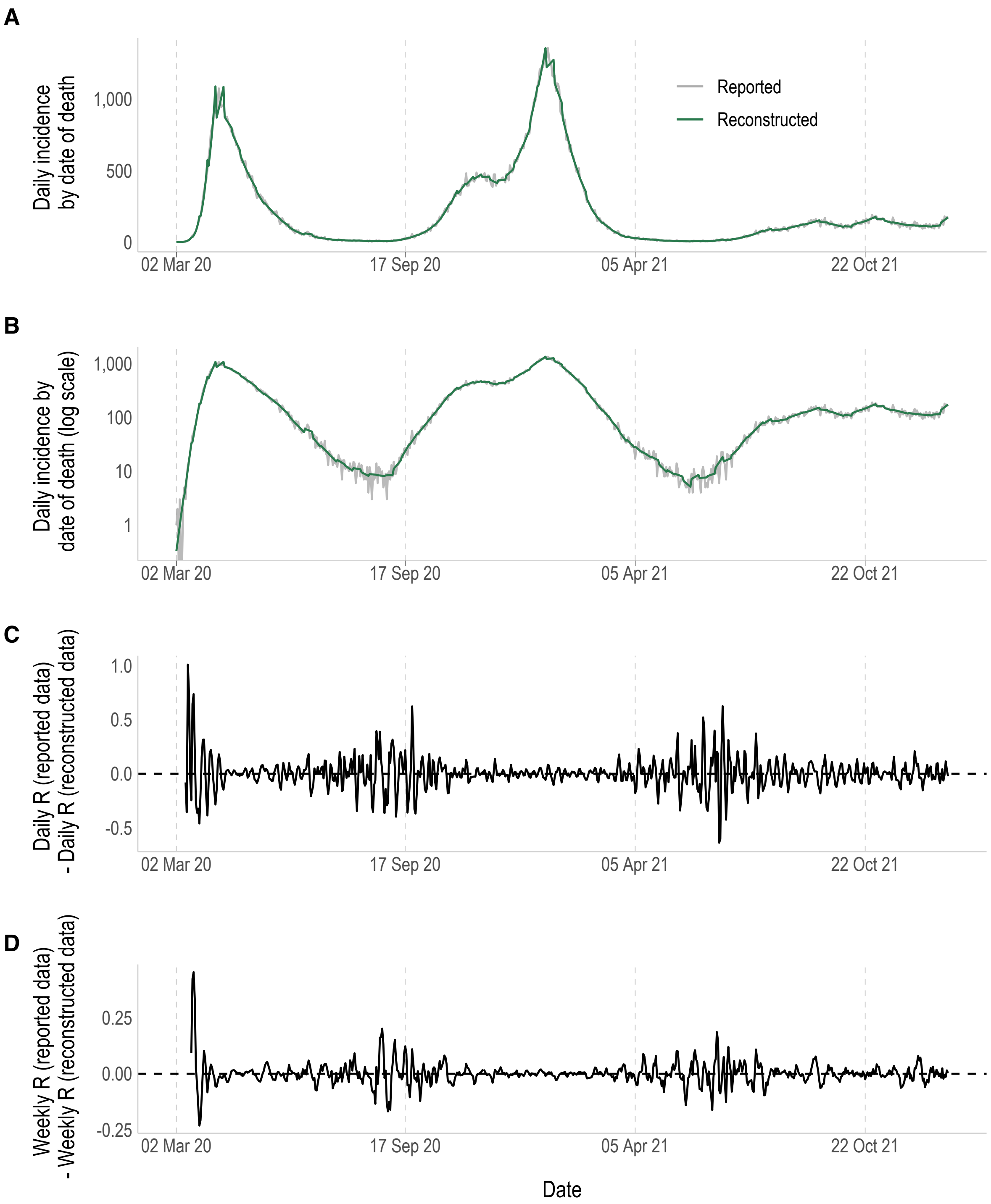

Figure S6. A-B) The reported (grey) and reconstructed (green) daily incidence of COVID-19 deaths within 28 days of a positive test, either on a natural scale (A) or log scale (B). C-D) The absolute difference in the C) daily and D) weekly sliding R_t estimates made using the reported data and the reconstructed data. Note that the y-axis scale is different in panels C and D.

d. Reported incidence patterns by weekday

There is a clear pattern in the reporting of influenza and COVID-19 cases, with generally higher incidence reported on Mondays, which then declines throughout the week, with the lowest incidence on the weekends (Figure S7A-B). Reported incidence appears lower on Fridays for influenza, however this dataset encompasses Christmas Day 2009 and New Year's

Day 2010, which both fell on Friday. On the other hand, COVID-19 deaths did not appear to show any pattern in reporting, with no clear deviations from the weekly mean incidence regardless of the weekday (Figure S7C).

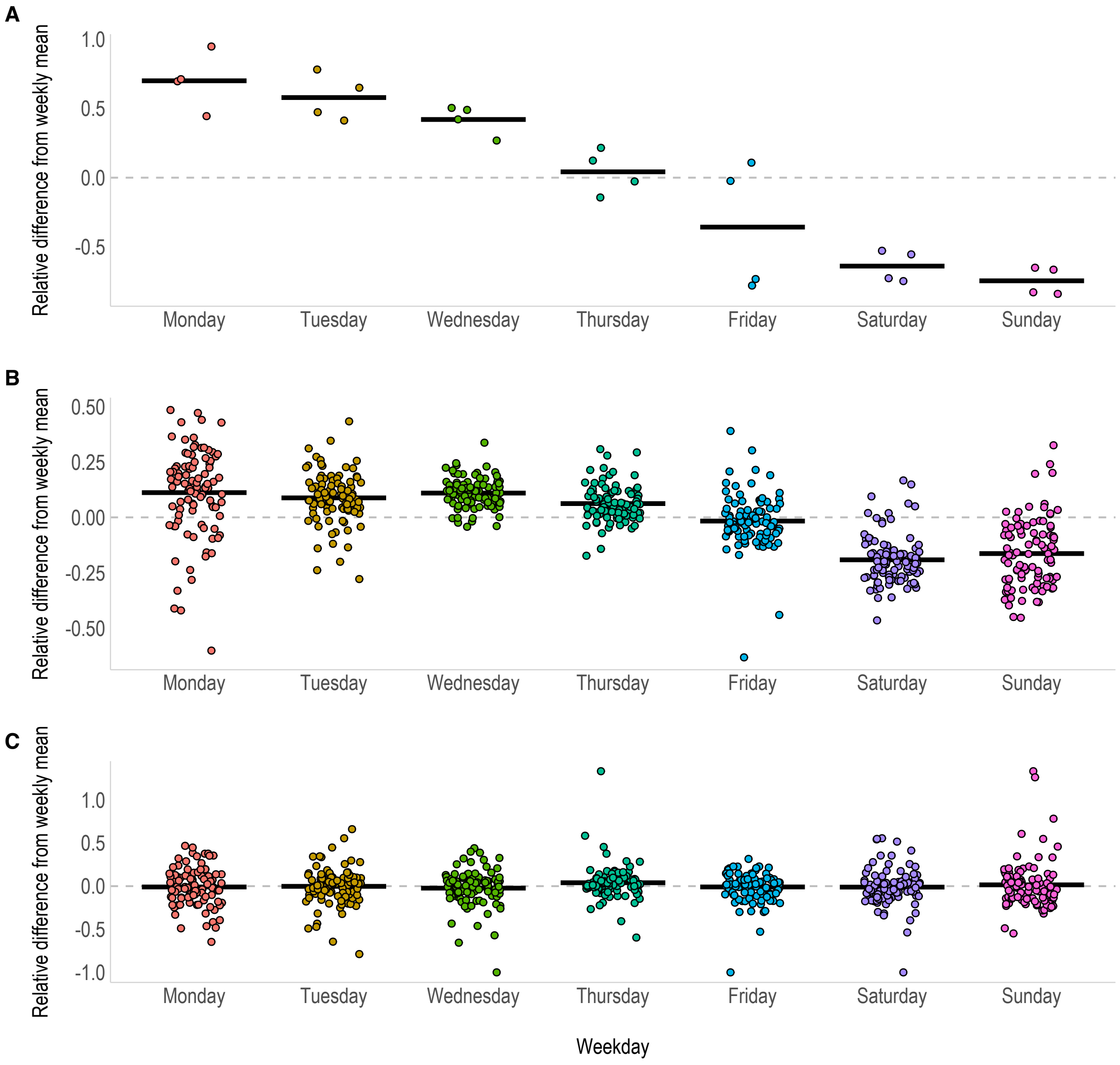

Figure S7. The relative difference between the reported incidence for A) influenza, B) COVID-19 cases and C) COVID-19 deaths, on each weekday compared to the mean incidence for that week. Only data for full calendar weeks are included. Black lines represent the mean relative difference and the dashed grey line indicates zero (corresponding to no difference between the reported incidence on that day compared to the weekly mean).

e. Classification tables

We directly compared the classification of the epidemic as increasing (95% credible interval of the R_t estimate is above 1), uncertain (95% credible interval encompasses 1), and declining (95% credible interval is below 1), depending on whether the R_t estimates were made using the reported or reconstructed daily data (Figure S8). The overall agreement in the classification of daily R_t estimates for influenza, COVID-19 cases, and COVID-19 deaths, was 44.4%, 74.4%, and 85.8% respectively. The overall agreement in the classification of weekly sliding R_t estimates was higher for each, with 81.8%, 94.9% and 93.3% agreement respectively. There is a greater correlation between the classification of estimates made using COVID-19 death data, reflected by the dark blue diagonal line across the grid squares. This is likely due to the reported deaths being less affected by weekend effects (see section 2d), and therefore the reported and reconstructed data (and the R_t estimates made from them) are more similar (Figure S8E-F).

0% | 1-15% | 16-30% | 31-45% | 46-60% | 61-75% | 76-90% | 91-100%

Influenza

Daily R

A

Agreement of the 95% CrI for estimates using **reconstructed** daily data (*n=27*)	95% CrI for estimates using **reported** daily data (*n=27*): Above 1	Encompassing 1	Below 1
Above 1	43.8% (*n=7*)	0% (*n=0*)	27.3% (*n=3*)
Encompassing 1	25% (*n=4*)	0% (*n=0*)	27.3% (*n=3*)
Below 1	31.3% (*n=5*)	0% (*n=0*)	45.5% (*n=5*)
Total (n)	*16*	*0*	*11*

Weekly sliding R

B

Agreement of the 95% CrI for estimates using **reconstructed** daily data (*n=22*)	95% CrI for estimates using **reported** daily data (*n=22*): Above 1	Encompassing 1	Below 1
Above 1	77.8% (*n=7*)	100% (*n=1*)	0% (*n=0*)
Encompassing 1	11.1% (*n=1*)	0% (*n=0*)	8.3% (*n=1*)
Below 1	11.1% (*n=1*)	0% (*n=0*)	91.7% (*n=11*)
Total (n)	*9*	*1*	*12*

COVID-19 cases

C

Agreement of the 95% CrI for estimates using **reconstructed** daily data (*n=650*)	95% CrI for estimates using **reported** daily data (*n=650*): Above 1	Encompassing 1	Below 1
Above 1	80.7% (*n=205*)	35.5% (*n=11*)	21.9% (*n=80*)
Encompassing 1	5.5% (*n=14*)	12.9% (*n=4*)	2.7% (*n=10*)
Below 1	13.8% (*n=35*)	51.6% (*n=16*)	75.3% (*n=275*)
Total (n)	*254*	*31*	*365*

D

Agreement of the 95% CrI for estimates using **reconstructed** daily data (*n=650*)	95% CrI for estimates using **reported** daily data (*n=650*): Above 1	Encompassing 1	Below 1
Above 1	96.2% (*n=255*)	40% (*n=8*)	2.5% (*n=9*)
Encompassing 1	1.5% (*n=4*)	35% (*n=7*)	0.3% (*n=1*)
Below 1	2.3% (*n=6*)	25% (*n=5*)	97.3% (*n=355*)
Total (n)	*265*	*20*	*365*

COVID-19 deaths

E

Agreement of the 95% CrI for estimates using **reconstructed** daily data (*n=643*)	95% CrI for estimates using **reported** daily data (*n=643*): Above 1	Encompassing 1	Below 1
Above 1	81.8% (*n=157*)	6.7% (*n=18*)	0% (*n=0*)
Encompassing 1	18.2% (*n=35*)	91.1% (*n=246*)	17.7% (*n=32*)
Below 1	0% (*n=0*)	2.2% (*n=6*)	82.3% (*n=149*)
Total (n)	*192*	*270*	*181*

F

Agreement of the 95% CrI for estimates using **reconstructed** daily data (*n=643*)	95% CrI for estimates using **reported** daily data (*n=643*): Above 1	Encompassing 1	Below 1
Above 1	98.3% (*n=226*)	3.5% (*n=6*)	0% (*n=0*)
Encompassing 1	1.7% (*n=4*)	86.0% (*n=147*)	6.2% (*n=15*)
Below 1	0% (*n=0*)	10.5% (*n=18*)	93.8% (*n=227*)
Total (n)	*230*	*171*	*242*

Figure S8. Comparison of the percentage agreement in the classification of R_t estimates as above 1 (lower bound of the 95% CrI is above 1), encompassing 1, or below 1 (upper bound of the 95% CrI is below 1), when using reported and reconstructed incidence data. Daily and weekly sliding R_t estimates are compared for influenza cases (A-B), COVID-19 cases (C-D), and COVID-19 deaths (E-F). Due to low incidence, the first 30 days of data is excluded for both COVID-19 datasets. Darker shades of blue correspond to greater percentage agreement, with a strong correlation represented by a dark diagonal line across the grid squares from top left to bottom right.

f. Computational time

All real data scenarios took less than 3 seconds to run on MacOS (2 GHz Quad-Core Intel Core i5) 16GB RAM (Table S1). The influenza cases scenario, with >57,000 cases, took 2 seconds to run. The COVID-19 cases and deaths scenarios, which estimated R_t over 97 and 96 weeks of incidence data, took 3 seconds to run.

Table S1. Time taken to estimate R_t in each of the real data scenarios.

Real data scenario	Length of time period analysed	Total incidence	Estimation time
Influenza cases	5 weeks	57,351	2s
COVID-19 cases	97 weeks	13,139,522	3s
COVID-19 deaths	96 weeks	149,557	3s

3. Simulation study

The simulation study involved assessing the performance of the method in multiple epidemic contexts. These included scenarios where R_t remains constant over time, or where R_t varies over time, with either a sudden stepwise change or a gradual change. For each scenario, 100 epidemic trajectories were stochastically generated using the R package projections.[3] Each epidemic was seeded with 7 days of 10 daily cases and then simulated over 70 days (10 weeks) using a renewal equation based branching process model, where incidence follows a Poisson distribution (eq. 1).

$$I_t \sim Poisson\left(R_t \sum_{s=1}^{t-1} I_s\, \omega_{t-s}\right) \tag{1}$$

The values of R_t estimated from the simulated incidence data were evaluated in terms of their bias, uncertainty and 95% coverage (Table S2).

Table S2. Definitions for the criteria used to assess the performance of our method in the simulation study.

Criterion	Definition
Bias	Absolute difference between the mean R_t estimate across the 100 simulations and the true value of R_t that the simulated incidence is based on.
Uncertainty	The mean width (across the 100 simulations) of the 95% credible interval for R_t estimates.
95% coverage	The proportion of R_t estimates across the 100 simulations with the true value of R_t within the 95% credible interval.

a. Constant R_t

For a scenario where R_t remains constant over time, four values of R_t were considered: 1, 1.25, 1.5 and 1.75. The R_t estimates all recovered the true value that the simulations were based on, with very little difference between the estimates made using the true daily and reconstructed daily data (Figure S9A-D). There was no evidence of bias in the estimates (Figure S9E-H), and as expected, the uncertainty declined over time as the number of cases rose (Figure S9I-L). When R_t was 1, as the seed for the simulated incidence trajectories is relatively low, some of the stochastic trajectories “died out” with case numbers falling to zero, which makes uncertainty appear to increase. The 95% coverage was consistently high (Figure S9M-P) and on average the true value of R_t was encompassed by the 95% credible interval 96-97% of the time in each scenario. However, the wavy pattern in these plots is likely to correspond to discontinuities in the reconstructed incidence data (section 3b, Figure S10).

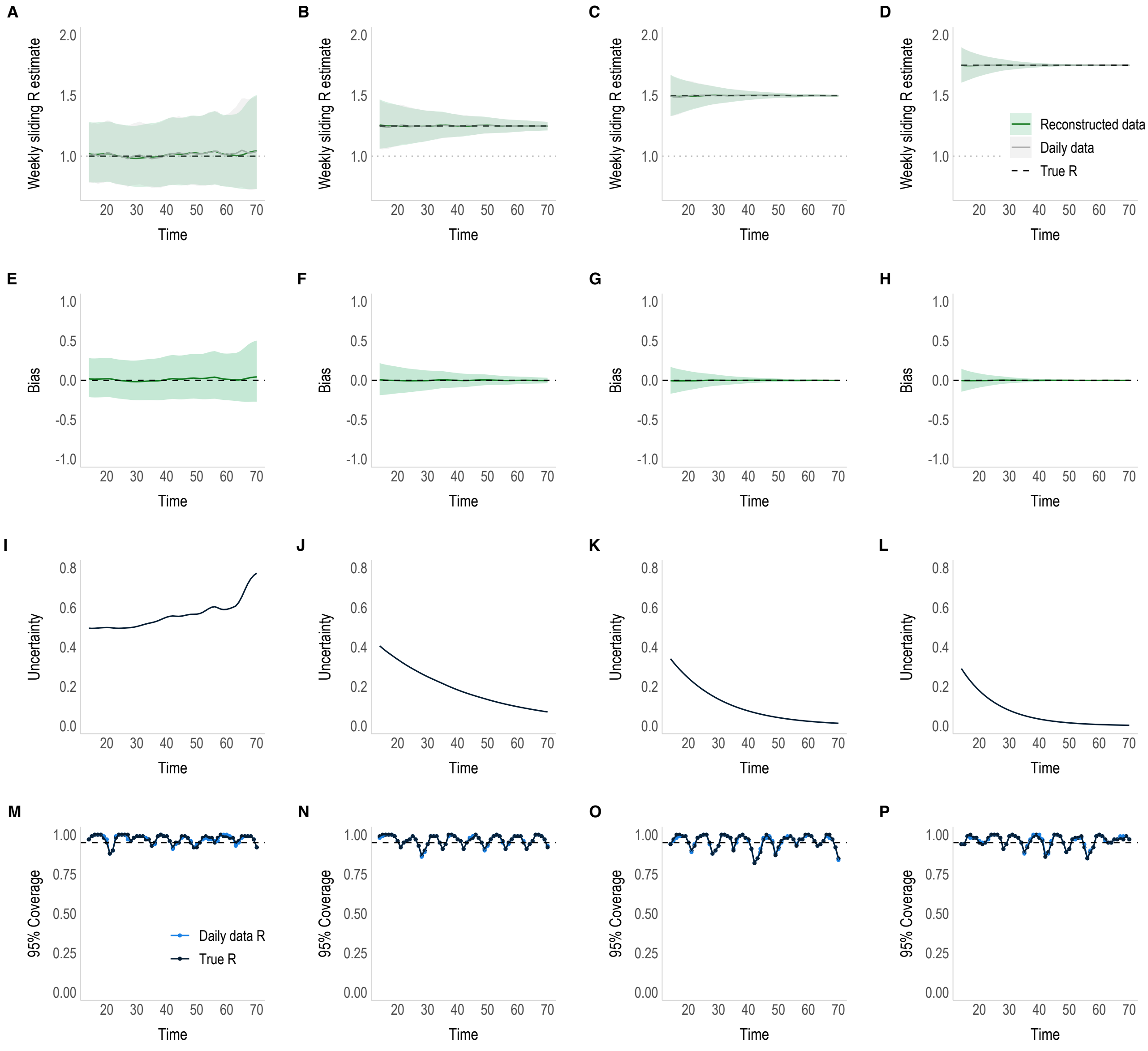

Figure S9. Performance of the method when estimating a constant R_t for 100 simulated epidemics. A-D) The mean weekly sliding R_t estimates using daily data (grey) and reconstructed data (green). The black dashed line represents the true value of R_t that the 100 simulations are based on, which is either A) 1, B) 1.25, C) 1.5, or D) 1.75. The estimates and their 95% credible intervals (shaded areas) are very similar and therefore overlap. E-H) The bias (or absolute difference) in the mean R_t estimated from reconstructed data compared to the true value of R_t. The shaded area corresponds to the 95% quantiles of the bias across the 100 simulations. I-L) The uncertainty (mean width of the 95% credible interval) in the R_t values estimated from reconstructed data. M-P) The proportion of the 100 R_t estimates obtained from reconstructed data where the 95% credible interval encompasses either the true value of R_t (black) or the value of R_t that would have been estimated from daily data (blue). Here, the 95% coverage is similar for both the true value of R_t and the daily data estimate, and therefore they overlap.

b. Discontinuities in the reconstructed incidence data

As part of the process of reconstructing daily incidence data from aggregated data, the reconstructed incidence is slightly adjusted using a constant (k_w) to ensure that if you were to re-aggregate it, it would still match the original aggregated data used as the input. This can result in discontinuities in the borders between time periods that the data has been aggregated over, which means that the reconstructed incidence is not completely smooth, even when there is no noise in the data (Figure S10). When R_t is estimated using sliding windows, these may align perfectly with the time windows that data were aggregated over or they may encompass the border between aggregations, and therefore the discontinuity in the data. As

shown in Figure S9M-P, this can lead to a wavy pattern in the 95% coverage, where the sliding window moves across the incidence data and can be more/less affected by the discontinuities depending on where they fall. This is important to be aware of, but as seen in the real data scenarios (section 1), reported daily data is often dramatically affected by intra-weekly variations in reporting, such as weekend effects. The reconstructed data, even with these discontinuities, is much smoother than the daily data that is typically reported. Nevertheless, we suggest that the sliding time window used to estimate R_t should be equal to or longer than the length of the aggregation window, to limit the effect of the discontinuities on the estimates.

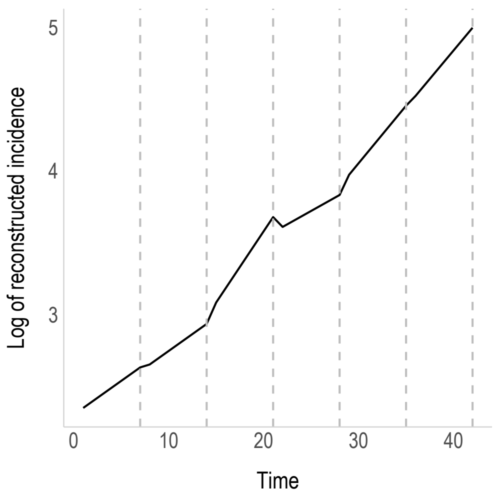

Figure S10. An example of reconstructed daily incidence data (on the log scale) with clear discontinuities on the borders between time periods where the data had been aggregated (grey dashed lines).

c. Time varying R_t: sudden change

In the first scenario for time varying transmissibility, we considered a stepwise decrease or increase in the value of R_t. R_t may suddenly decrease or increase following the rapid implementation or relaxation of stringent control measures, for instance, a strict mass 'lockdown' event.

We considered four decreasing scenarios, where on day 35 of the simulated outbreak, R_t falls from: 1.25 to 0.75, 1.25 to 1, 1.5 to 1.25, or 1.75 to 1.5. The true value of R_t was successfully recovered in all scenarios, except for a week-long delay following the step change (Figure S11A-D), which corresponds to dips in the bias plots (Figure S11E-H). This delay is due to the weekly sliding window used for R_t estimation, which would encompass incidence data before and after the step change.

As expected, uncertainty declines as case numbers rise when $R_t > 1$ (Figure S11I-L). The decrease in R_t from 1.25 to 0.75 replicates a situation where control measures successfully bring R_t below 1, resulting in falling case numbers and increased uncertainty after day 35 (Figure S11I). Similarly, when R_t falls from 1.25 to 1, case numbers become stable, leading to a plateau in the uncertainty (Figure S11J).

Importantly, despite not recovering the true value of R_t in the week following the step change, a high proportion of the estimates recovered the value of R_t that would have been estimated from the reported daily data (Figure S11M-P). There is a slightly larger drop in the 95% coverage when R_t falls from 1.75 to 1.5 (Figure S11P), however, this is due to higher case numbers in this scenario resulting in very narrow 95% credible intervals. Overall, the 95% credible interval encompassed the true value of R_t 85-90% of the time, which rose to 95-97% if the week following the step change was excluded.

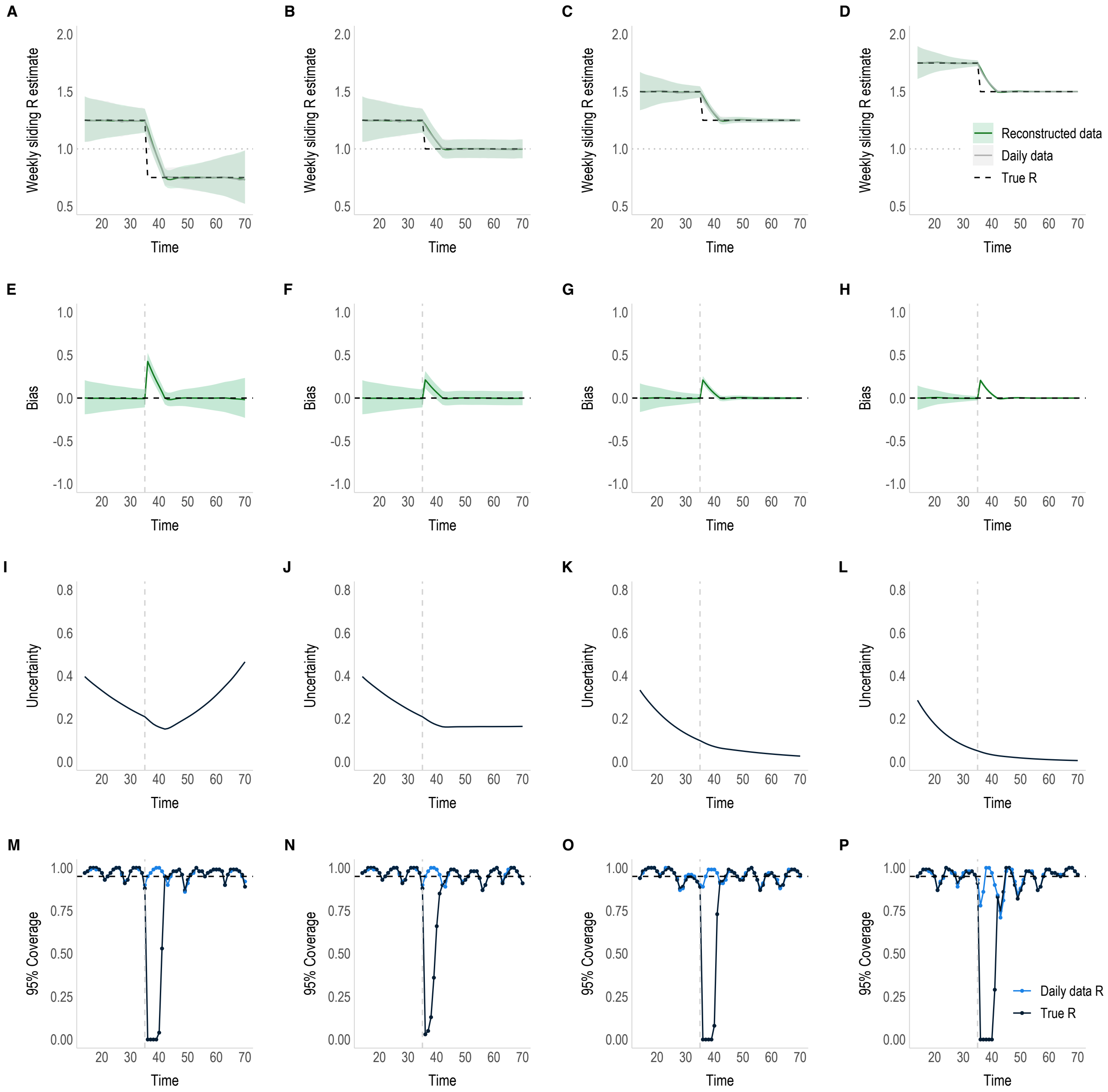

Figure S11. Performance of the method when estimating a time varying R_t for 100 simulated epidemics. In this scenario, R_t suddenly decreases on day 35 (grey dashed line) and remains constant before and after the step change. A-D) The mean weekly sliding R_t estimates using daily data (grey) and reconstructed data (green). The black dashed line represents the true value of R_t that the 100 simulations are based on, which is either a decrease from A) 1.25 to 0.75, B) 1.25 to 1, C) 1.5 to 1.25, or D) 1.75 to 1.5. The grey dotted line represents the threshold of $R_t = 1$. The estimates (plotted at the end of each time window) and their 95% credible intervals (shaded area) are very similar and therefore overlap. E-H) The bias (or absolute difference) in the mean R_t estimated from reconstructed data compared to the true value of R_t. The shaded area corresponds to the 95% quantiles of the bias across the 100 simulations. I-L) The uncertainty (mean width of the 95% credible interval) in the R_t values estimated from reconstructed data. M-P) The proportion of the 100 R_t estimates made using reconstructed data where the 95% credible interval encompasses either the true value of R_t (black) or the value of R_t that would have been estimated from daily data (blue).

A sudden stepwise increase in R_t is perhaps more difficult to explain in a real-world context, but it is also shown here for the sake of completion. Three scenarios for a stepwise increase in R_t were considered: 1 to 1.25, 1.25 to 1.5, and 1.5 to 1.75. Similarly, the true value of R_t was well recovered in each scenario, but there was a week-long delay following the

step change (Figure S12A-F). The uncertainty was stable when R_t is 1 and falls as case numbers rise (Figure S12G-I). Finally, despite the drop in 95% coverage for the true value of R_t, the estimates from reconstructed data recovered what would have been estimated from simulated daily data (Figure S12J-L). Across these scenarios, the 95% credible interval encompassed the true value of R_t 87-96% of the time, rising to 95-97% if the week following the step change is excluded.

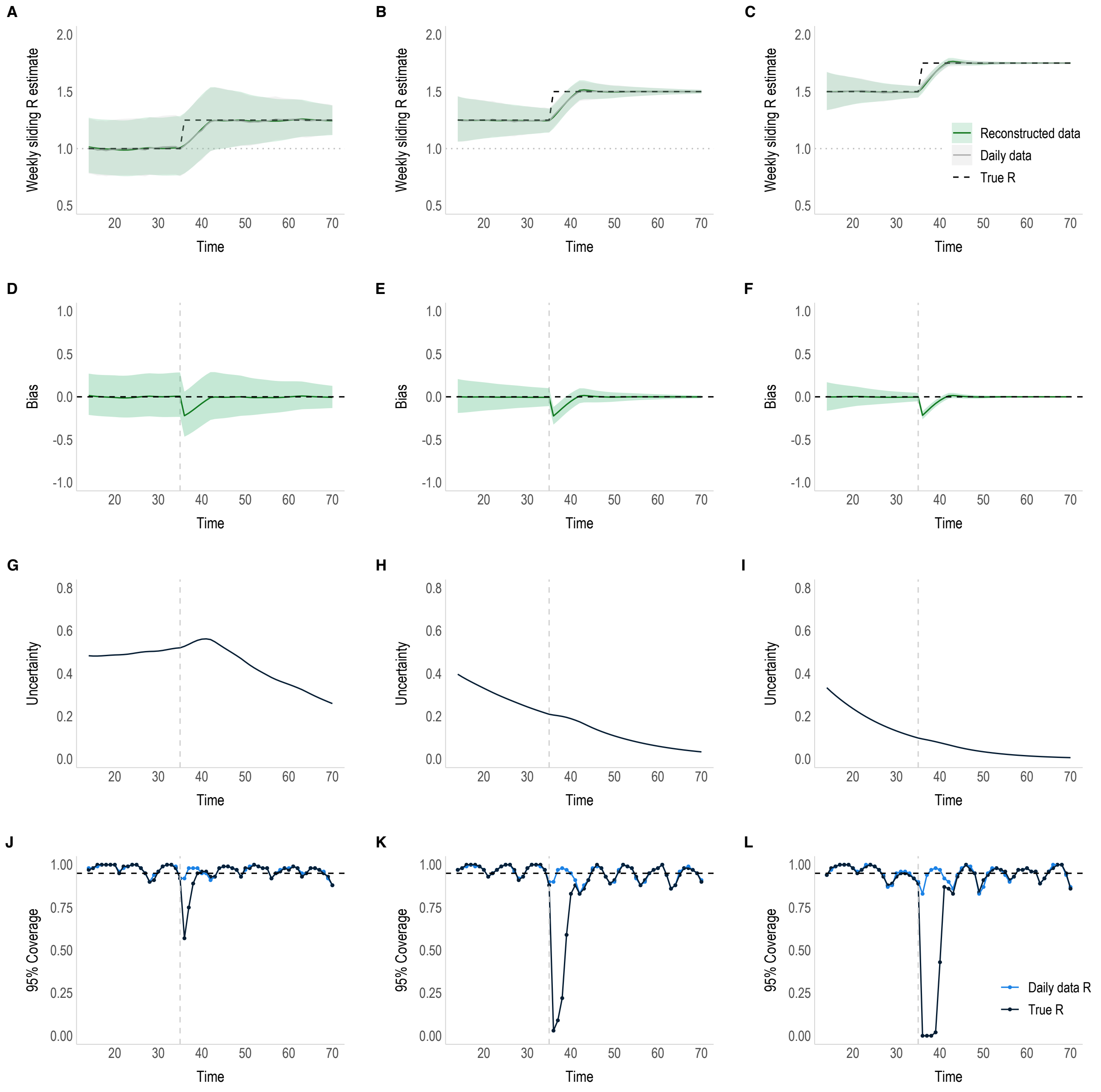

Figure S12. Performance of the method when estimating a time varying R_t for 100 simulated epidemics. In this scenario, R_t suddenly increases on day 35 (grey dashed line) and remains constant before and after the step change. A-C) The mean weekly sliding R_t estimates using simulated daily data (grey) and reconstructed data (green). The black dashed line represents the true value of R_t that the 100 simulations are based on, which is either an increase from A) 1 to 1.25, B) 1.25 to 1.5, or C) 1.5 to 1.75. The grey dotted line represents the threshold of $R_t = 1$. The estimates (plotted at the end of each time window) and their 95% credible intervals (shaded area) are very similar and therefore overlap. D-F) The bias (or absolute difference) in the mean R_t estimated from reconstructed data compared to the true value of R_t. The shaded area corresponds to the 95% quantiles of the bias across the 100 simulations. G-I) The uncertainty (mean width of the 95% credible interval) in the R_t values estimated from reconstructed data. J-L) The proportion of the 100 R_t estimates made

using reconstructed data where the 95% credible interval encompasses either the true value of R_t (black) or the value of R_t that would have been estimated from daily data (blue).

d. Time-varying R_t: gradual change

Changes in transmissibility are often more gradual over time. For example, R_t could slowly decrease as a population becomes more aware of a circulating pathogen and modifies their behaviour accordingly, or R_t could increase in response to the gradual easing of restrictions, such as social distancing measures, or a gradual decline in compliance to such restrictions. In the following scenarios, we considered a gradual change in R_t that occurred over the course of 30 days, starting on day 20 and ending on day 50. Four scenarios were considered for a gradually decreasing R_t, where R_t falls from: 1.25 to 0.75, 1.25 to 1, 1.5 to 1.25, and 1.75 to 1.5 (Figure S13). Three scenarios were considered for a gradually increasing R_t, where R_t increases from: 1 to 1.25, 1.25 to 1.5, and 1.5 to 1.75.

As above, during the period of gradual change, the estimates were affected by the lag due to the weekly sliding window for R_t estimation (Figure S13A-D & Figure S14A-C). This means that R_t was slightly overestimated during the 30 days that R_t was gradually decreasing and slightly underestimated when R_t was gradually increasing (Figure S13E-H & Figure S14D-F). The uncertainty declined as case numbers rose (Figures S13I-L & S14G-I), increased when R_t fell below 1 (Figure S13I) and plateaued when $R_t = 1$ (Figures S13J & S14G). The lag in the estimates caused some notable drops in the 95% coverage (Figures S13M-P & S14J-L). This is particularly prominent when incidence is high and credible intervals are small, such as in the gradual decrease in R_t from 1.75 to 1.5 scenario, where the true value of R_t was encompassed by the 95% credible interval 61% of the time (Figure S13P). For all other gradually decreasing scenarios and the gradually increasing scenarios, true R_t was recovered between 80-94% and 77-96% of the time respectively. It is important to note, however, that even when substantial dips in coverage occurred, the bias remained small, meaning there was very little difference between the estimate and the true value of R_t despite not being exactly the same (Figure S13E-H).

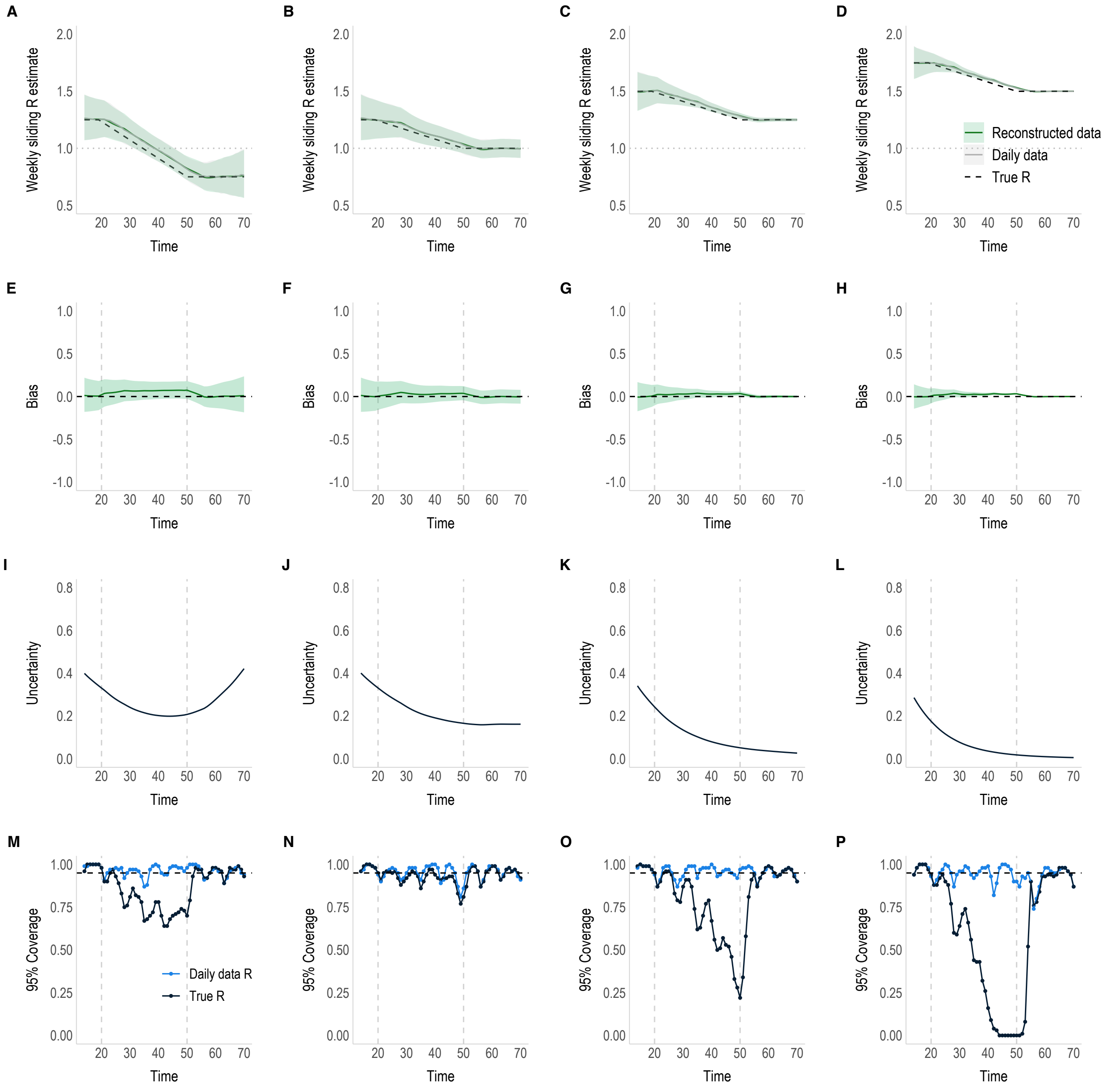

Figure S13. Performance of the method when estimating a time varying R_t for 100 simulated epidemics. In this scenario, R_t gradually decreases over the course of 30 days (day 20 to day 50, shown with grey dashed lines), and remains constant before and after the change. A-D) The mean weekly sliding R_t estimates using daily data (grey) and weekly data (green). The black dashed line represents the true value of R_t that the 100 simulations are based on, which is either a decline from A) 1.25 to 0.75, B) 1.25 to 1, C) 1.5 to 1.25, or D) 1.75 to 1.5. The grey dotted line represents the threshold of $R_t = 1$. The estimates (plotted at the end of each time window) and their 95% credible intervals (shaded area) are very similar and therefore overlap. E-H) The bias (or absolute difference) in the mean R_t estimated from weekly data compared to the true value of R_t. The shaded area corresponds to the 95% quantiles of the bias across the 100 simulations. I-L) The uncertainty (mean width of the 95% credible interval) in the R_t values estimated from weekly data. M-P) The proportion of the 100 R_t estimates made using weekly data where the 95% credible interval encompasses either the true value of R_t (black) or the value of R_t that would have been estimated from daily data (blue).

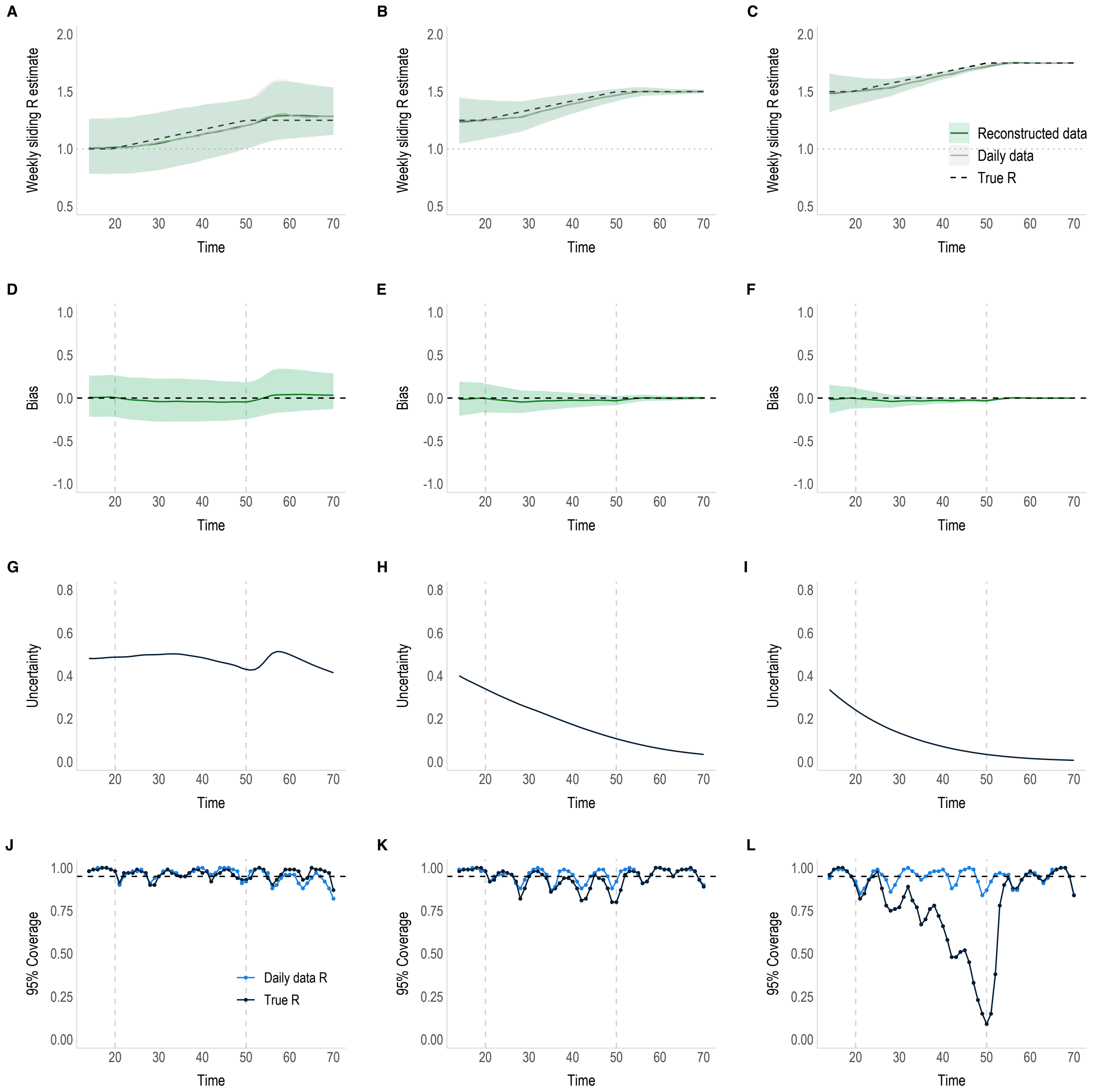

Figure S14. Performance of the method when estimating a time varying R_t for 100 simulated epidemics. In this scenario, R_t gradually increases over the course of 30 days (day 20 to day 50, shown with grey dashed lines), and remains constant before and after the change. A-C) The mean weekly sliding R_t estimates using daily data (grey) and weekly data (green). The black dashed line represents the true value of R_t that the 100 simulations are based on, which is either an increase from A) 1 to 1.25, B) 1.25 to 1.5, or C) 1.5 to 1.75. The grey dotted line represents the threshold of $R_t = 1$. The estimates (plotted in the middle of each time window) and their 95% credible intervals (shaded area) are very similar and therefore overlap. D-F) The bias (or absolute difference) in the mean R_t estimated from weekly data compared to the true value of R_t. The shaded area corresponds to the 95% quantiles of the bias across the 100 simulations. G-I) The uncertainty (mean width of the 95% credible interval) in the R_t values estimated from weekly data. J-L) The proportion of the 100 R_t estimates made using weekly data where the 95% credible interval encompasses either the true value of R_t (black) or the value of R_t that would have been estimated from daily data (blue).

e. **Influence of R_t plotting time relative to the time window used for estimation**

By default, the EpiEstim R package estimates R_t over weekly sliding time windows ending at time t, at which point R_t is plotted. An alternative to this, is to plot R_t in the middle of the time window, centred around t. This would mean that R_t estimates cannot be made in real-time, i.e., if a weekly window was used you can only estimate R_t at t + 3.5. However, the advantage is that retrospective R_t estimates would be less influenced by the lag corresponding to the length of the time window.

To demonstrate this, we reconsider the scenarios with a gradual and sudden change in R_t. When gradual changes in R_t are plotted in the middle of the time window (t-3.5), the true value of R_t is well recovered and the 95% coverage is considerably improved in comparison to estimates plotted at the end of the time window (Figure S15). Overall, the 95% credible intervals of the gradual R_t estimates plotted at the mid-point of the time window encompassed the true value of R_t 94-97% and 88-96% of the time for the increasing and decreasing scenarios respectively (compared to 77-96% and 61-94% when R_t is plotted at the end of the time window). In the gradual change from 1.5 to 1.75 and 1.75 to 1.5 scenarios, there is still a dip in coverage which occurs when case numbers are so high that the 95% credible intervals become extremely narrow (Figure S15C & J). Therefore, when the sliding window encompasses incidence data before R_t plateaus, it means R_t is slightly under- or overestimated for increasing and decreasing R_t respectively. However, it is evident that the mean estimates are negligibly different to the true value, despite not being exactly the same.

In the stepwise change in R_t scenarios, there is also some improvement in 95% coverage (Figure S16). There is still a lag corresponding to the length of the time window, but now the change in R_t is detected earlier and the dip in coverage is centred around the time change on day 35. As above, the dips in coverage are larger when case numbers are very high, and the 95% credible intervals are small. The 95% credible interval of estimates plotted at the mid-point of the time window encompassed the true value of R_t 88-96% and 85-93% of the time for stepwise increasing and decreasing scenarios respectively, which is a modest improvement compared to 87-96% and 85-90% when R_t is plotted at the end of the time window.

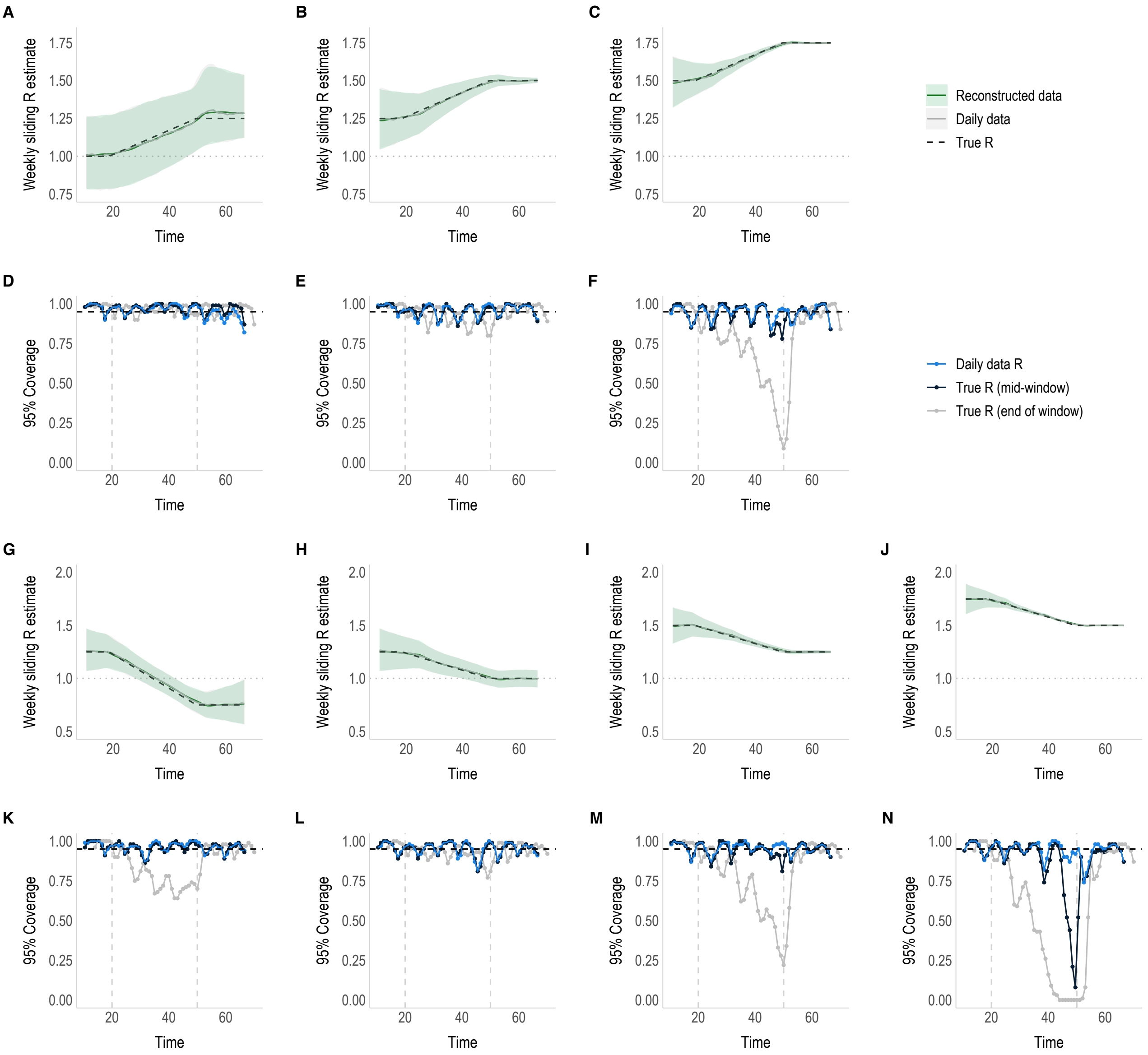

Figure S15. Plotting the gradual change in R_t scenarios for 100 simulated epidemics in the middle of the time window. Here, the gradually increasing (A-C) and decreasing (G-J) R_t estimate plots are exactly as in figures S13 and S14, except they are plotted at t-3.5. Each 95% coverage plot (D-F & K-N) corresponds to the plot directly above and shows the proportion of the 100 R_t estimates made using weekly data where the 95% credible interval encompasses either the true value of R_t (black) or the value of R_t that would have been estimated from daily data (blue). As a reference, the 95% coverage of the true value of R_t for the R_t estimates plotted at the end of the time window (see figures S13 & S14) are shown in grey.

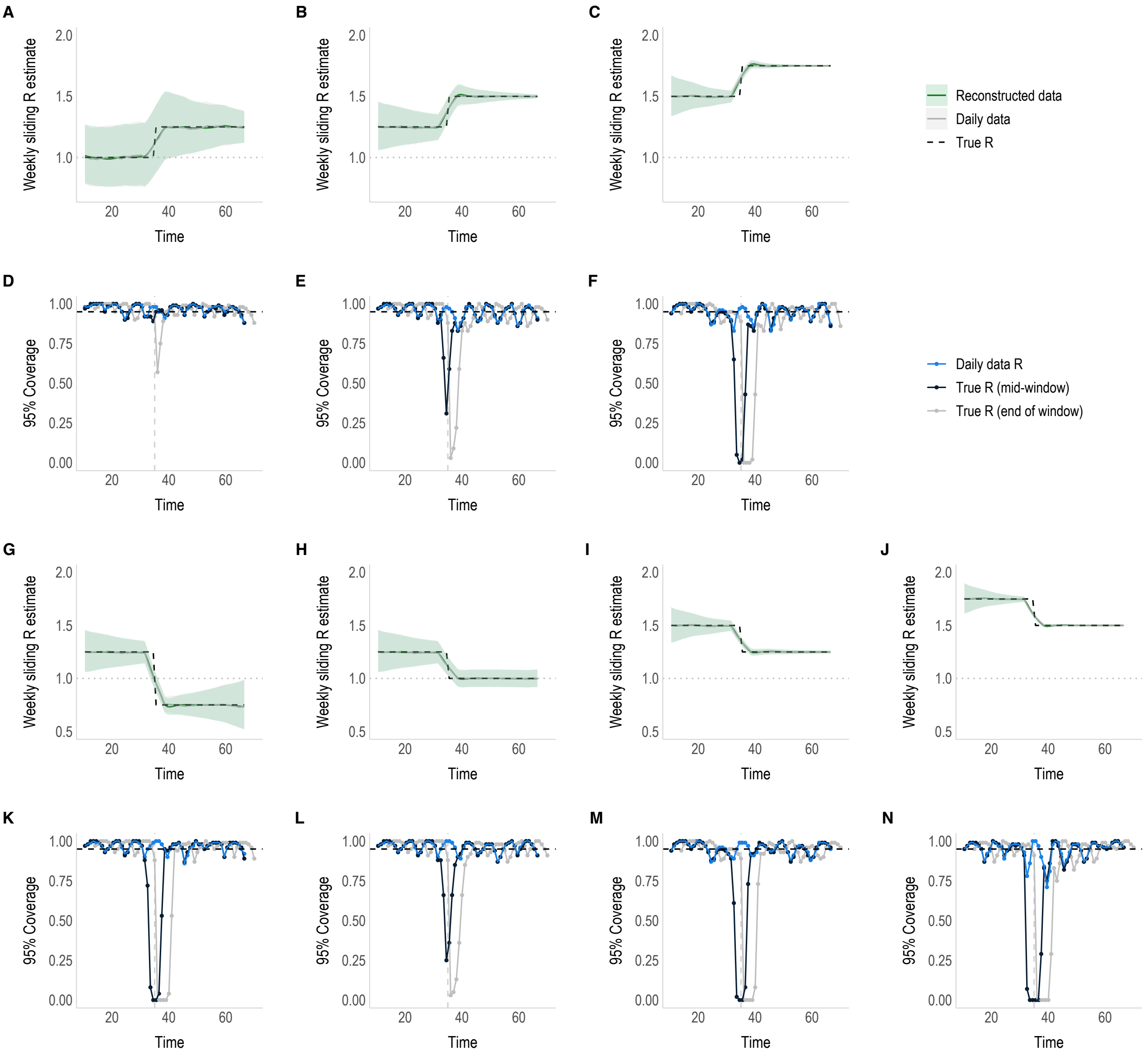

Figure S16. Plotting the stepwise change in R_t scenarios for 100 simulated epidemics in the middle of the time window. Here, the stepwise increasing (A-C) and decreasing (G-J) R_t estimate plots are exactly as in figures S11 and S12, except they are plotted at t-3.5. Each 95% coverage plot (D-F & K-N) corresponds to the plot directly above and shows the proportion of the 100 R_t estimates made using weekly data where the 95% credible interval encompasses either the true value of R_t (black) or the value of R_t that would have been estimated from daily data (blue). As a reference, the 95% coverage of the true value of R_t for the R_t estimates plotted at the end of the time window (see figures S11 & S12) are shown in grey.

f. Weekend effects

To mimic incidence data with weekend effects, the simulated incidence for scenarios where R_t remained constant at 1.5, suddenly decreased from 1.5 to 1.25, and gradually increased from 1.25 to 1.5, were further modified so that 80% of cases were taken off the final two days of each aggregation window and redistributed uniformly over the first two days of the aggregation window (Figure S17A-C). This appears roughly similar to the pattern observed in the influenza case study (Figure S7). R_t was then estimated using daily (Figure S17D-F), weekly (Figure S17G-I), and two-weekly (Figure S17J-L) sliding windows, using the simulated data (with weekend effects) and the reconstructed daily data.

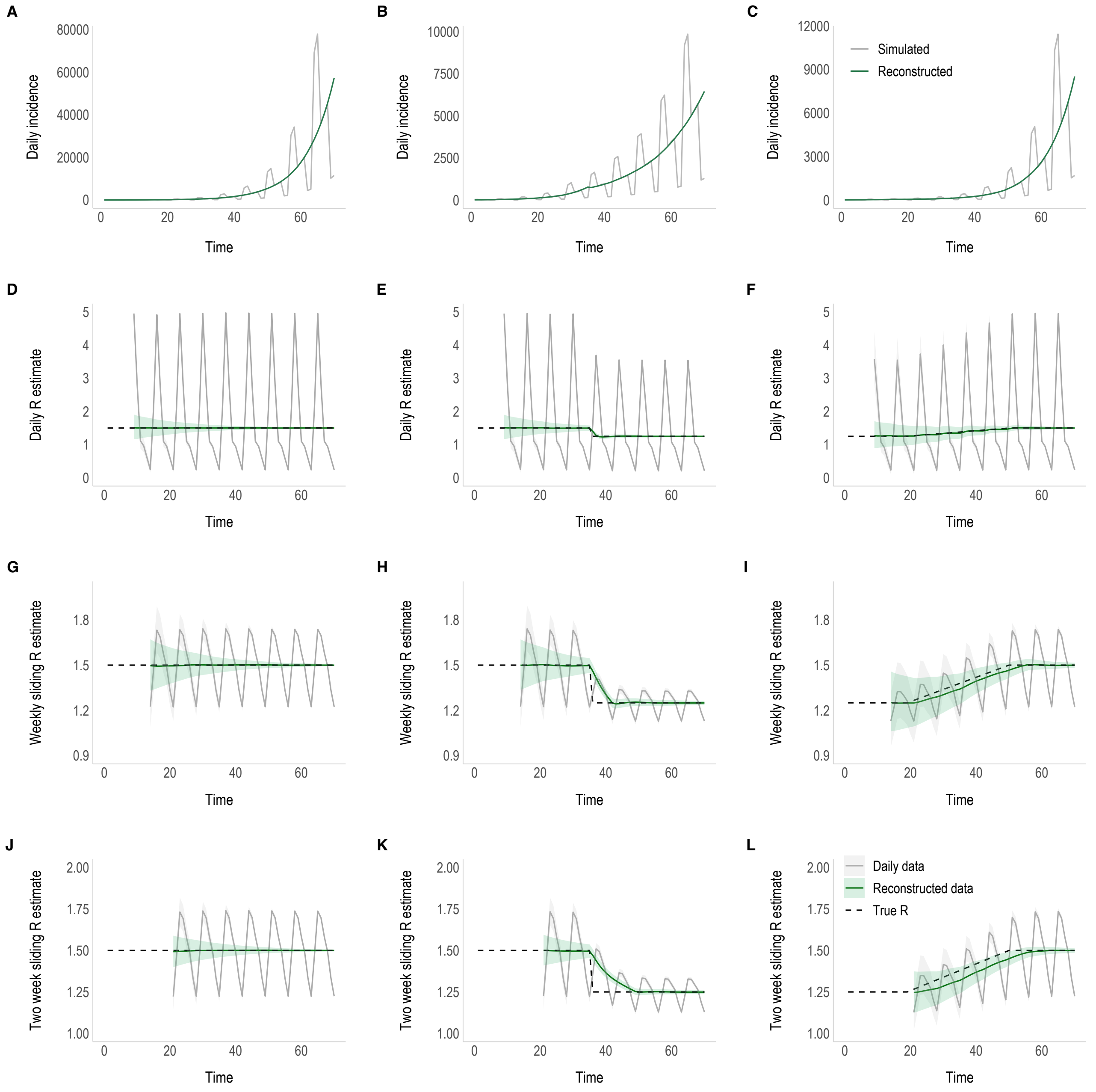

Figure S17. Assessing the performance of our method in the presence of weekend effects in the reported data. A-C) Example of reported (grey) and reconstructed (green) daily incidence data taken from one of the 100 simulated epidemics (selected at random) for a (A) constant R_t of 1.5, (B) stepwise decrease in R_t from 1.5 to 1.25, and (C) gradual increase in R_t from 1.25 to 1.5. R_t was estimated over D-F) daily, G-I) weekly, and J-L) two-weekly sliding time windows. The black dashed line is the true value of R_t that the simulated data was based on. Note: y-axis limits for each incidence plot varies and the y-axis scale is different for the daily R_t estimate plots (second row).

For every scenario, the R_t estimates were considerably smoother and more accurate when the incidence had been reconstructed from weekly data. This is simply because the reconstruction smoothed out the intra-weekly variability and removed the effect of the noise from R_t estimates.

g. Mid-aggregation variations in transmissibility

Although the approach can successfully smooth out noise in the data due to phenomena such as weekend effects in reporting, this also means that genuine variations in transmissibility that occur mid-aggregation window would not be

captured. To demonstrate this, we simulate incidence data where transmissibility either increases from 1.25 to 1.5, or decreases from 1.5 to 1.25, on weekends (Figure S18). This may occur if contact patterns change on the weekends, e.g. transmission may increase at the weekends due to larger group gatherings or events, or decrease if transmission is limited to households rather than workplaces.

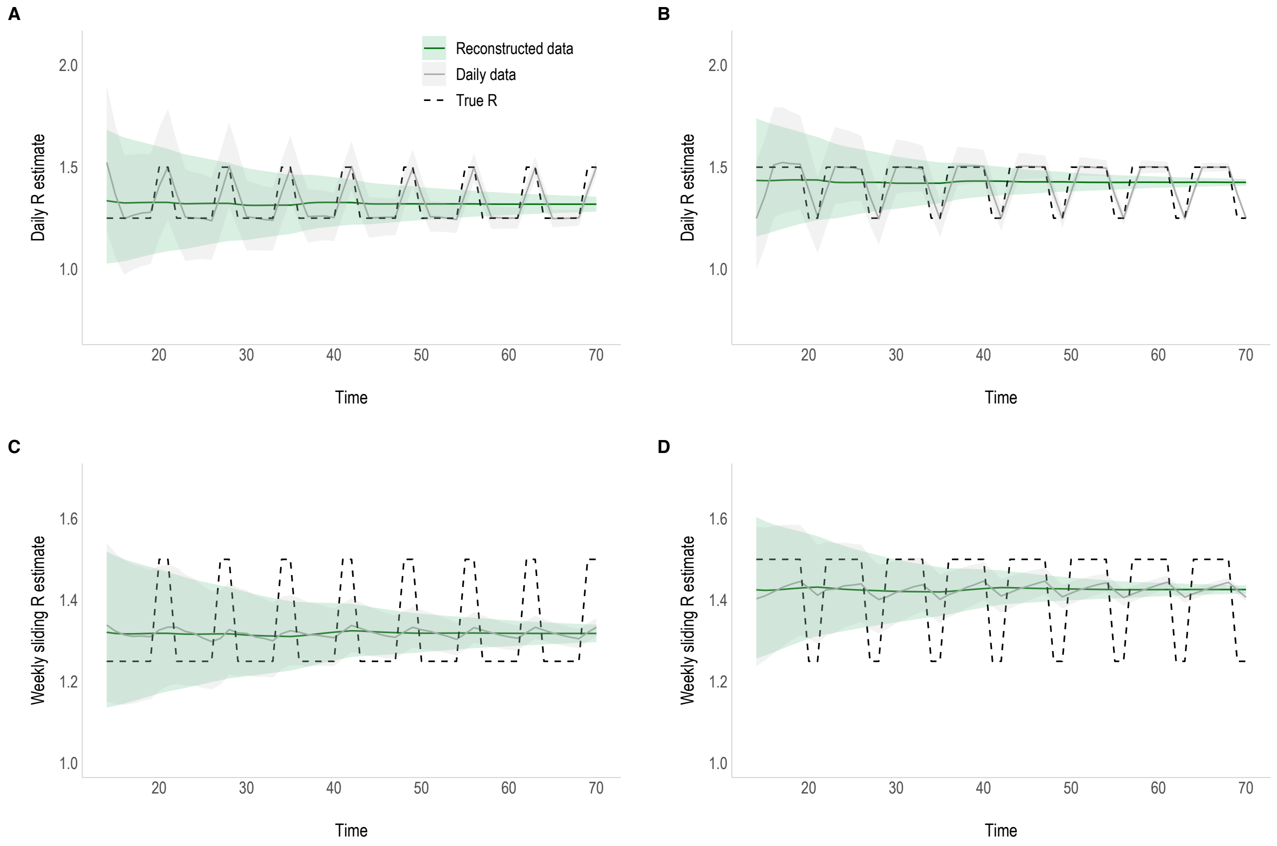

Figure S18. Assessing the ability to detect genuine mid-aggregation window variations in transmissibility (either increasing (left), or decreasing (right) on weekends) from reconstructed daily data (green) and the true simulated daily data (grey), with R_t estimated over daily (A-B) or weekly-sliding (C-D) time windows.

When R_t is estimated over daily time windows (Figure S18A-B) variations in transmissibility are well captured by R_t estimates obtained from the simulated daily data, but completely smoothed out when using the reconstructed daily data. When R_t is estimated over weekly-sliding time windows (Figure S18C-D), R_t estimates from the simulated daily data are considerably smoothed out, but small temporal variations are still apparent, whilst they disappear in R_t estimates using the reconstructed daily data.

In a real-world context, it can be difficult to disentangle administrative noise in reporting, such as weekend effects, from these genuine patterns in transmission. Therefore, there is a trade off in temporal smoothing between removing noise and detecting important variations in transmissibility that have epidemiological significance.

h. Number of iterations

For each scenario, the EM algorithm used to reconstruct the incidence (and in turn estimate R_t) converged over a small number of iterations, with negligible differences beyond 5 iterations (Figure S19). Given that the computational time is so short (see section 2f), the default number of iterations was set to 10 in the R package. However, a convergence check ensures that the final iteration of the reconstructed daily incidence does not differ from the previous iteration beyond a tolerance of 10^{-6}, and the number of iterations can be modified by the user.

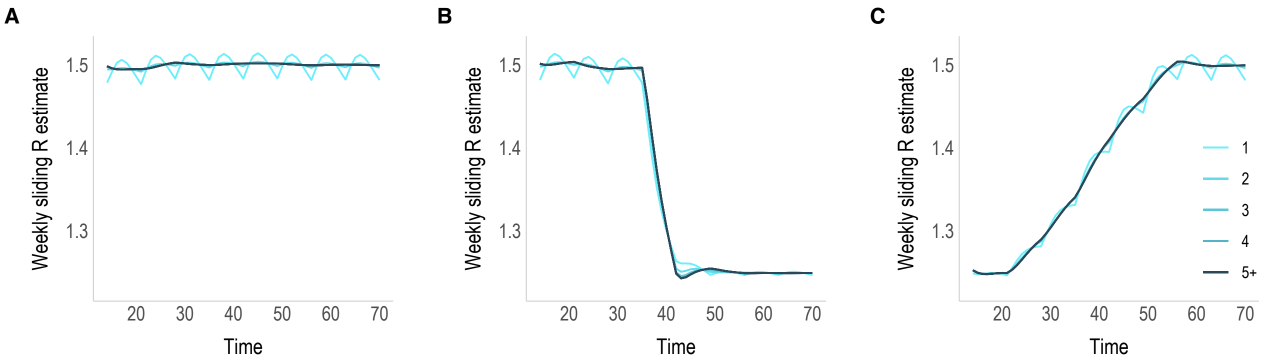

Figure S19. The mean weekly sliding R_t estimates generated after each of the 10 iterations from 100 stochastically simulated epidemics for the following scenarios: A) constant R_t of 1.5, B) stepwise decrease in R_t from 1.5 to 1.25, and C) gradual increase in R_t from 1.25 to 1.5.

i. Different temporal aggregations

In addition to weekly incidence, the method can be successfully applied to other temporal aggregations of data. Here, we showcase the method applied to data that has been aggregated to 3-day, 10-day and 14-day timescales. First, we consider the ideal scenario where the end of the aggregation window aligns perfectly with the R_t step-change on day 35 (Figure S20). Then we consider how the accuracy of the estimations would be affected if the step-change fell in the middle of the aggregation window (Figure S21).

When the aggregation window is aligned with the step-change, there is only the usual lag in detecting temporal changes in R_t, corresponding to the width of the sliding time window used for the estimation (Figure S20 B, E, H & K). However, when aggregation windows misalign with the step-change, there is a further lag (Figure S21 H & K). This is because, in the process of daily incidence reconstruction, the growth rate is assumed constant within each aggregation window. When aggregation windows misalign with the step-change, the growth rate is essentially smoothed out when the daily incidence is reconstructed. This is important to be aware of when using larger aggregations of data, as the loss of some temporal resolution in R_t estimates will be unavoidable.

As mentioned in section 2b, we recommend that the user ensures that the sliding window used to estimate R_t is equal to or longer than the length of the aggregation window. Although a longer sliding window leads to a further lag in R_t estimates for the stepwise change in R_t scenarios (Panels H & K in Figures S20 & S21), the R_t estimates generated are smoother. This is because they are less affected by discontinuities in the reconstructed incidence data, the result of which can be clearly seen in the gradual change in R_t scenarios (Panels I & L in Figures S20 & S21), where estimates made using 7-day sliding windows appear wavy.

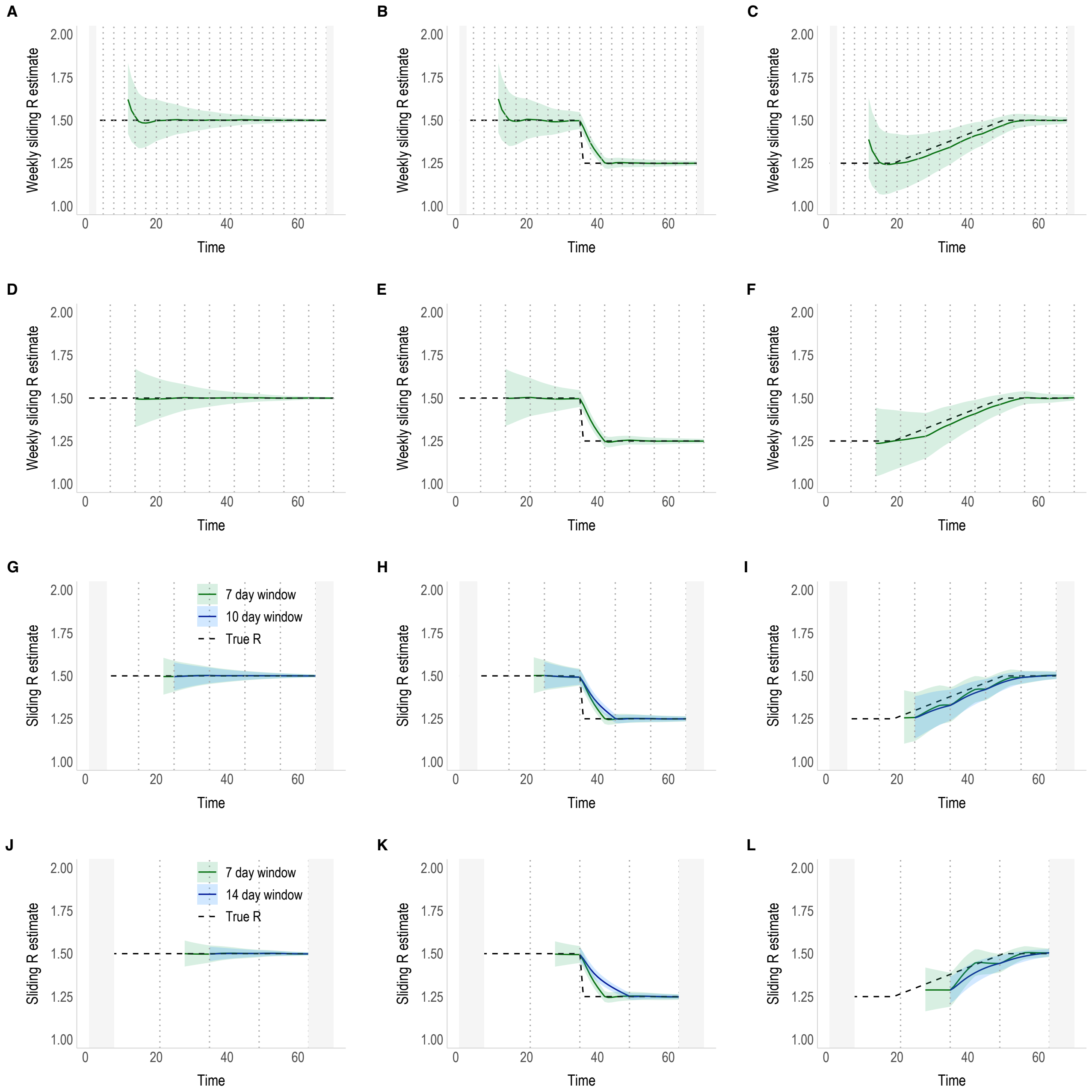

Figure S20. Performance of the method using alternative aggregations of incidence data when the end of aggregation windows <u>align</u> with the step change on day 35. We consider the following scenarios: a constant R_t of 1.5 (left), stepwise decrease in R_t from 1.5 to 1.25 (middle), and a gradual increase in R_t from 1.25 to 1.5 (right), with data aggregated over A-C) 3 days, D-F) 7 days, G-I) 10 days, and J-L) 14 days. For the 10-day and 14-day aggregations of data, we compare estimates made using weekly sliding windows (green) with sliding windows of length matching that of the aggregation of data (blue). For all scenarios, R_t estimates are plotted at the end of the sliding time window. Grey dotted lines show the end of each aggregation window and the grey shaded areas are the time periods excluded in order to align the aggregation windows (if necessary).

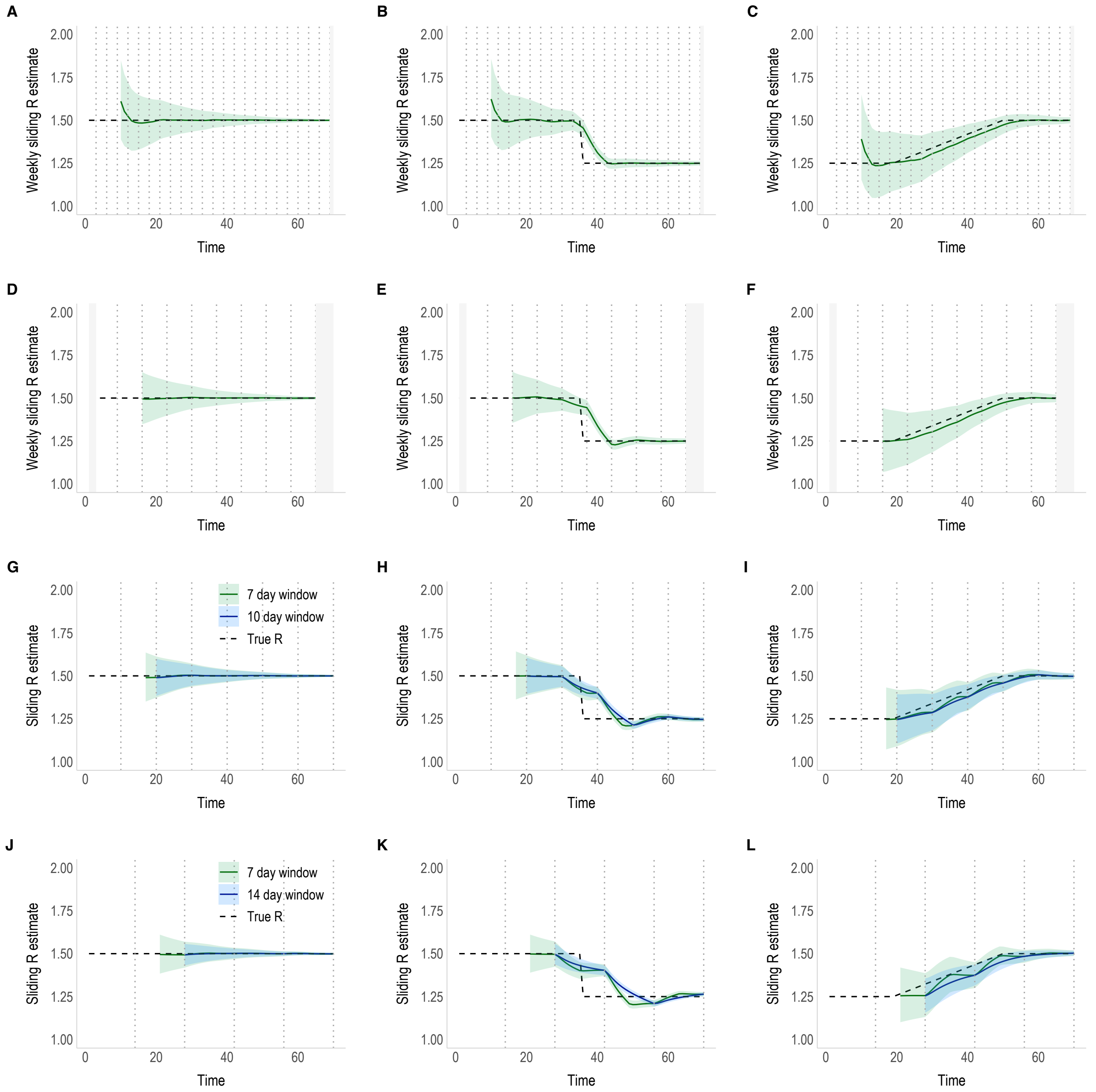

Figure S21. Performance of the method using alternative aggregations of incidence data when the end of aggregation windows do not align with the step change on day 35. We consider the following scenarios: a constant R_t of 1.5 (left), stepwise decrease in R_t from 1.5 to 1.25 (middle), and a gradual increase in R_t from 1.25 to 1.5 (right), with data aggregated over A-C) 3-days, D-F) 7-days, G-I) 10-days, and J-L) 14-days. For the 10-day and 14-day aggregations of data, we compare estimates made using weekly sliding windows (green) with sliding windows of length matching that of the aggregation of data (blue). For all scenarios, R_t estimates are plotted at the end of the sliding time window. Grey dotted lines show the end of each aggregation window and the grey shaded areas are the time periods excluded in order to misalign the aggregation windows (if necessary).

4. **Alternative approach**

Here, we compare our EM algorithm method to a locally estimated scatterplot smoothing (LOESS) approach, implemented using the 'loess()' function from the stats R package.[4]

LOESS fits a smooth curve through datapoints using local regression. It is not designed to disaggregate aggregated data, but rather it interpolates between observations to infer missing data. In addition, it can only interpolate between observations and not beyond them. For use here, we therefore had to make a few assumptions. To address the first issue, we fit LOESS curves to the naïve disaggregation of incidence for each week (e.g. 70 cases in a week / 7 days = 10 cases per day). We then need to decide which day of the week the "observation" is made on (acting as the smoothing points e.g. 10 cases observed on Monday), which influences which days are excluded from the reconstruction because they fall outside the range of the "observations". Finally, we need to decide which degree of the polynomials to be used (varying between 0, 1, and 2).

Using the influenza dataset, we consider two options for smoothing points, either that they fall at the end of the aggregation windows (days 7, 14, 21, 28, 35 - option A), or in the middle of the aggregation windows (days 3, 10, 17, 24, 31 - option B). We then fit the LOESS curves to those "observations" (Figure S22).

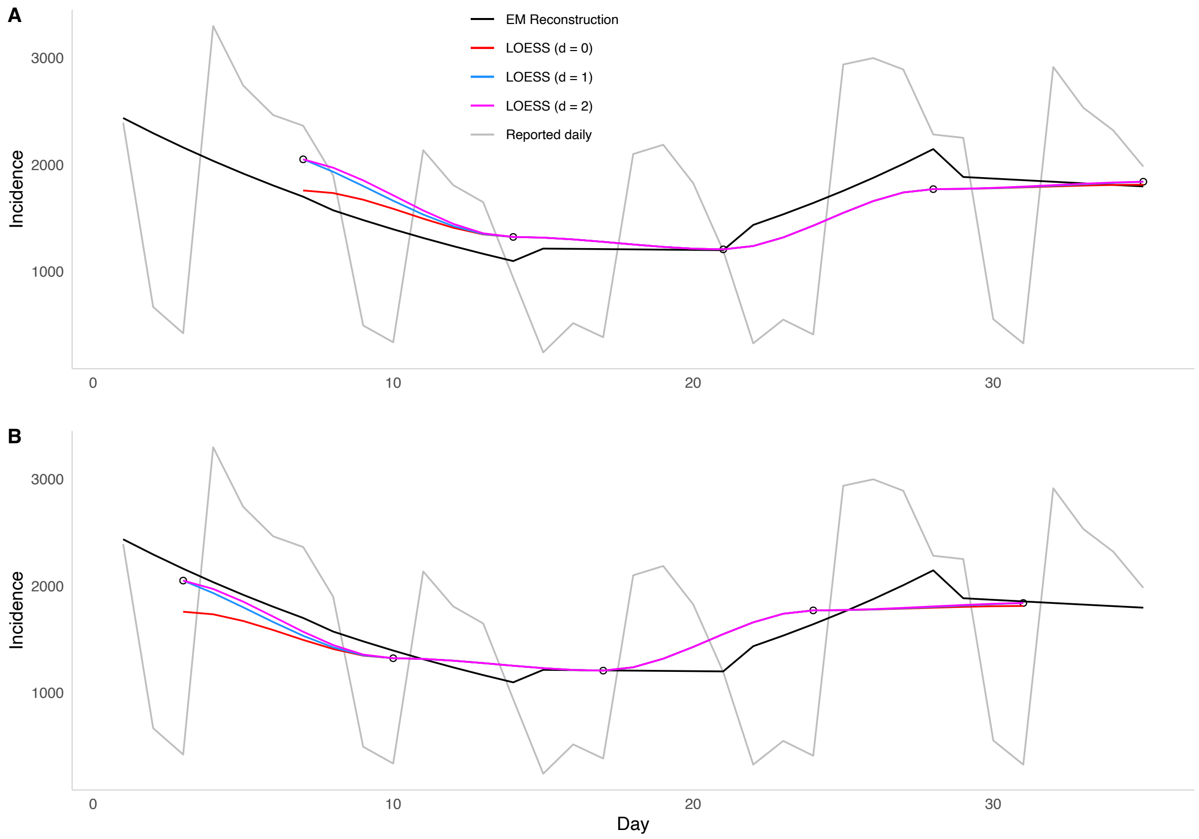

Figure S22. Comparison between the reconstructed daily incidence generated by the EM algorithm and LOESS smoothing, based on smoothing points (black circles) at either the end (A – days 7, 14, 21, 28 and 35) or middle (B - days 3, 10, 17, 24, 31) of aggregation time windows. The "observed" incidence at the smoothing points was computed as the naïve disaggregation of the weekly incidence for that week. The reported daily incidence of influenza (grey), the daily reconstructed incidence generated by the EM algorithm (black), and the daily reconstructed incidence generated by LOESS smoothing with the degree of the polynomial set to either 0 (red), 1 (blue), or 2 (magenta). Note that LOESS smoothing does not allow interpolation beyond the observations.

For option A, data in the first week cannot be reconstructed, whilst for option B, the first two days and final four days of incidence cannot be reconstructed. By using the aggregation mid-points as smoothing points, option B could be a fairer comparison to the incidence reconstructed by the EM algorithm, however, as incidence cannot be reconstructed up to the final day, this approach could not be used in real-time analysis.

As shown, LOESS smoothing generates smooth daily incidence curves without any discontinuities between aggregation windows (see section 3b). However, unlike LOESS, our method preserves the aggregated incidence totals supplied by the user. Table S3 illustrates the differences in the weekly totals when the reconstructed daily incidence generated by either the EM algorithm or by LOESS smoothing is re-aggregated.

Table S3. Differences between the re-aggregated reconstructed daily incidence data generated by either the EM algorithm method or LOESS smoothing. "True weekly" is the weekly aggregations of the reported daily influenza dataset that would be supplied by the user.

Incidence	Week				
	1	2	3	4	5
True weekly	14353	9264	8450	12399	12885
EM reconstruction	14353	9264	8450	12399	12885
Option A) Smoothing points at the end of time window					
LOESS (degree = 0)	NA	10570 (+1306)	8798 (+348)	10706 (-1693)	12565 (-320)
LOESS (degree = 1)	NA	11029 (+1765)	8798 (+348)	10706 (-1693)	12637 (-248)
LOESS (degree = 2)	NA	11237 (+1973)	8798 (+348)	10706 (-1693)	12656 (-229)
Option B) Smoothing points at the midpoint of time window					
LOESS (degree = 0)	NA	9228 (-36)	9186 (+736)	12308 (-91)	NA
LOESS (degree = 1)	NA	9247 (-17)	9186 (+736)	12320 (-79)	NA
LOESS (degree = 2)	NA	9275 (+11)	9186 (+736)	12331 (-68)	NA

The differences in the re-aggregated reconstructed daily incidence data are substantial with both options. Reaching up to 21% mismatch for option A and 9% for option B.

We then explored the extent to which these discrepancies in the reconstructed incidence may affect the reproduction number estimates (Figure S23). R_t estimates from the LOESS reconstructions do not follow the trend in R_t from the daily reported data as closely as R_t estimates from the reconstructed incidence obtained from the EM algorithm.

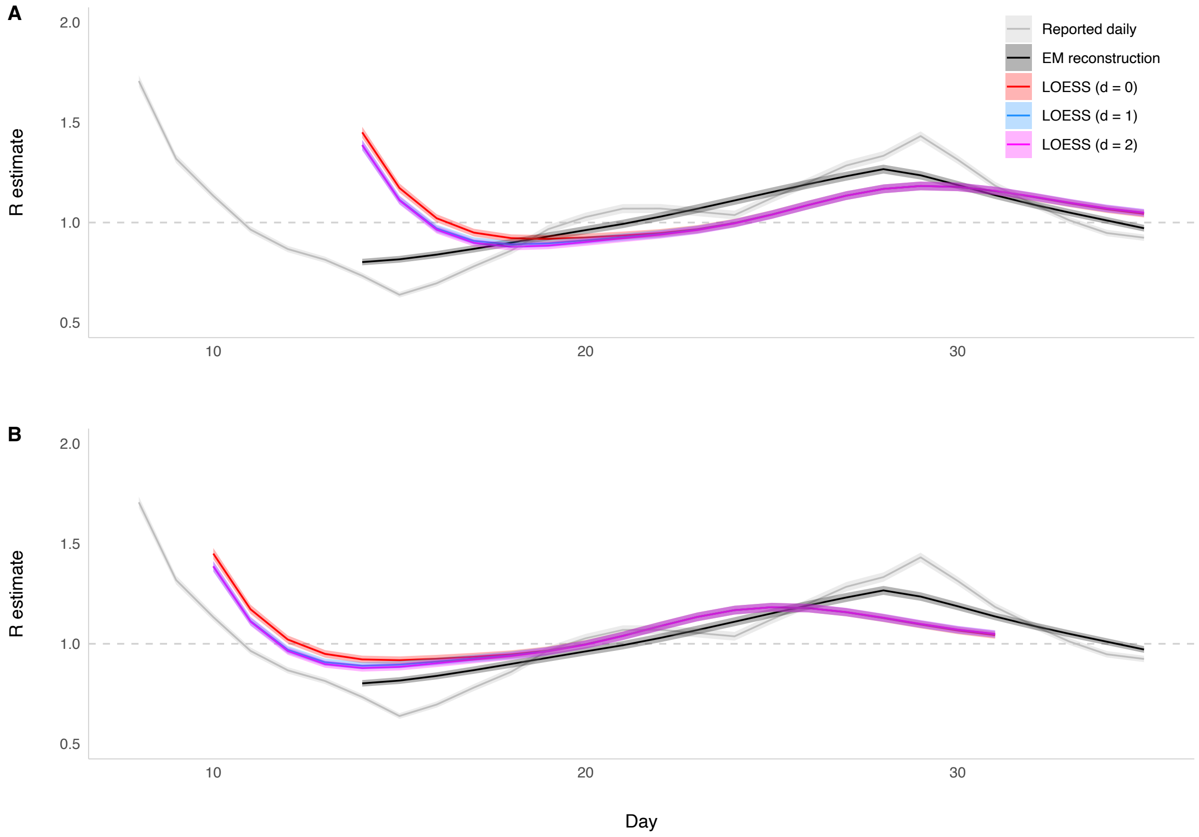

Figure S23. Comparison between R_t estimates made using reconstructed daily incidence generated by the EM algorithm (black), or by LOESS smoothing with the degree of the polynomial set to either 0 (red), 1 (blue), or 2 (magenta). The LOESS approach is based on smoothing points at the end (A) or middle (B) of aggregation time windows.

Bias in the R_t estimates was assessed by computing the absolute difference in estimates from the reported daily data and the reconstructed daily data, generated by the EM algorithm or by LOESS smoothing (Figure S24). Despite slightly better performance around day 20 with option B (Figure S24B), we show that estimates using LOESS reconstructions introduce more bias into R_t estimates than those estimated from incidence reconstructed by the EM algorithm. Therefore, we believe it is important to preserve the integrity of the data as much as possible in the reconstruction of incidence.

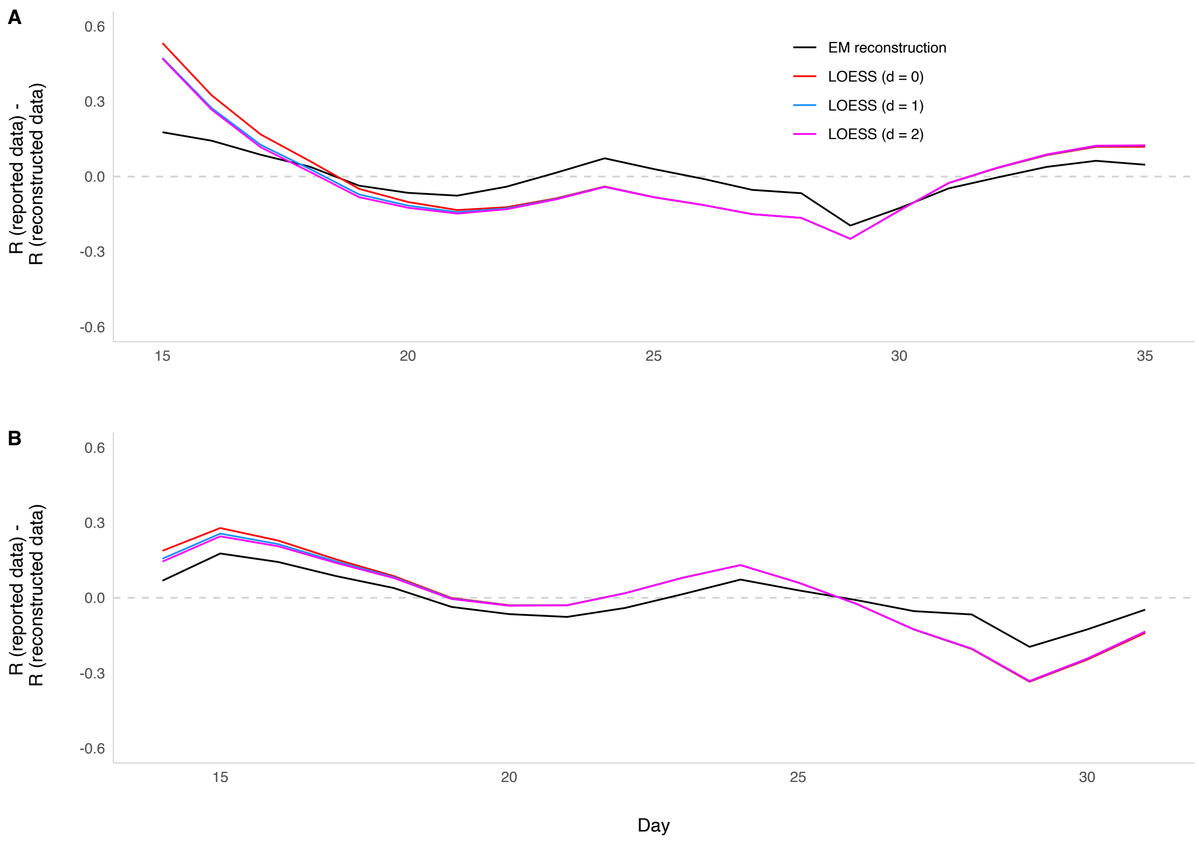

Figure S24. The absolute difference between R_t estimates made using the reported daily data and the reconstructed daily incidence, generated by the EM algorithm (black), or by LOESS smoothing with the degree of the polynomial set to either 0 (red), 1 (blue), or 2 (magenta). The grey dashed line represents an absolute difference of 0. The LOESS approach is based on smoothing points at the end (A) or middle (B) of aggregation time windows.

5. Zika virus disease

In order to consider a disease with non-respiratory transmission, we also present an example using real-world data for the vector-borne Zika virus disease. As no daily incidence data for Zika was available to us, weekly incidence of Zika in Rio de Janeiro, Brazil, from 14th November 2015 to 15th April 2016, was obtained from Ferguson et al 2016.[5] A workaround approach, that is commonly used when applying the original EpiEstim method to temporally aggregated data, involves supplying the SI at the same timescale as the weekly incidence data used. We compared our EM approach to this workaround, assuming a mean SI of 20 days and standard deviation of 7.4 days for the reconstructed incidence generated by the EM approach, and a mean SI of 2.86 weeks and standard deviation 1.06 weeks for the workaround method (Figure S25).[5]

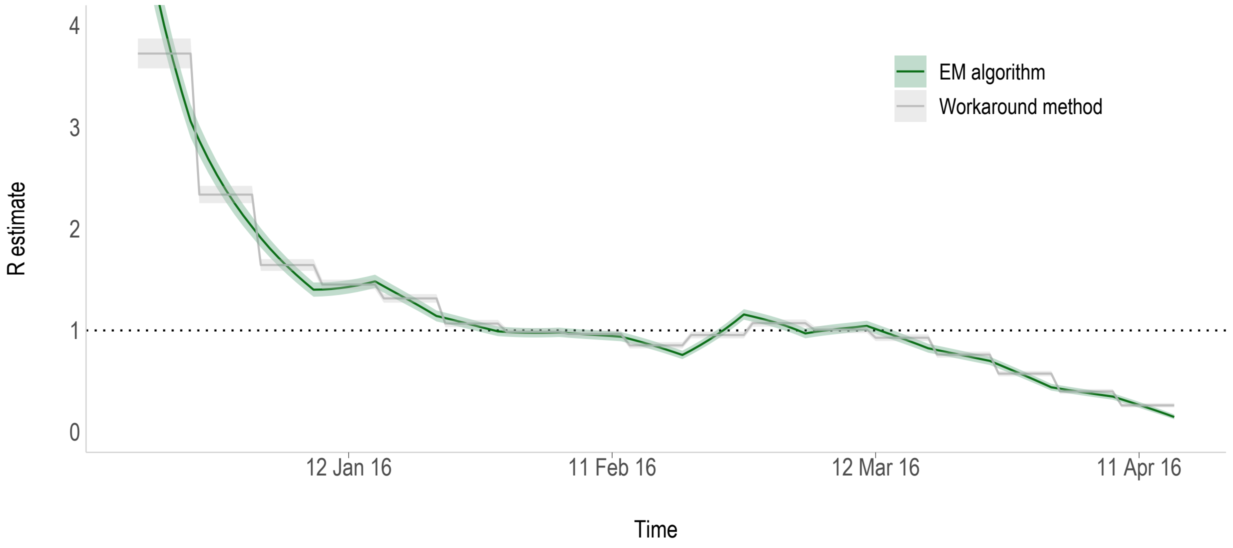

Figure S25. Comparison of R_t estimates obtained using a workaround approach (where a weekly SI is supplied to match the incidence data) and the EM approach. For clarity, we show the R_t estimates between 19th December 2015 and 15th April 2016, when R_t is estimated to have fallen below 4.

We demonstrate that the reconstructed incidence generated by the EM algorithm produces estimates that are consistent with those from the workaround method. However, as R_t can now be estimated from daily reconstructed incidence, the estimates have greater temporal resolution.

It is also important to note that this workaround method cannot be used if the serial interval is shorter than the time windows that data are aggregated over, further highlighting the benefit of our novel EM approach.

6. Alternative analysis pipelines

Our EM approach to reconstruct daily incidence data uses EpiEstim within the algorithm, and thus the framework of our method relies on the likelihood used in EpiEstim. In this paper, we focused on producing a consistent statistical framework for R_t estimation, however, it is also possible to use the reconstructed daily incidence generated by our EM algorithm in other analysis pipelines which use alternative methods for R_t estimation.

As an example, we present an analysis comparing R_t estimation using EpiEstim with an alternative R_t estimation method, EpiLPS,[6] using the same set of reconstructed daily incidence data. Using data from our influenza case study, we supply EpiLPS with the reconstructed incidence generated as an output of our EM approach and use the default EpiLPS parameters to then estimate R_t (Figure S26). EpiLPS estimates are highly influenced by the weekend effects in the reported data (Figure S26A), and more so than weekly EpiEstim R_t estimates (Figure S26B). However, as is the case with EpiEstim, the reconstructed incidence smooths out the oscillations in the EpiLPS R_t estimates (Figure S26A). We note that by default, EpiEstim plots R_t estimates at the end of the time window used, so to be able to fairly compare the R_t estimates generated by EpiEstim and EpiLPS, we shift the EpiLPS estimates by 3 days to compare the mid-points of estimation windows (Figure S26B). We demonstrate that the R_t estimates produced by both approaches are very similar, with wider credible intervals using EpiLPS. However, we note that unlike EpiEstim this approach uses data beyond time t for smoothing, and although it has great value in retrospective analysis, this is not a real-time approach.

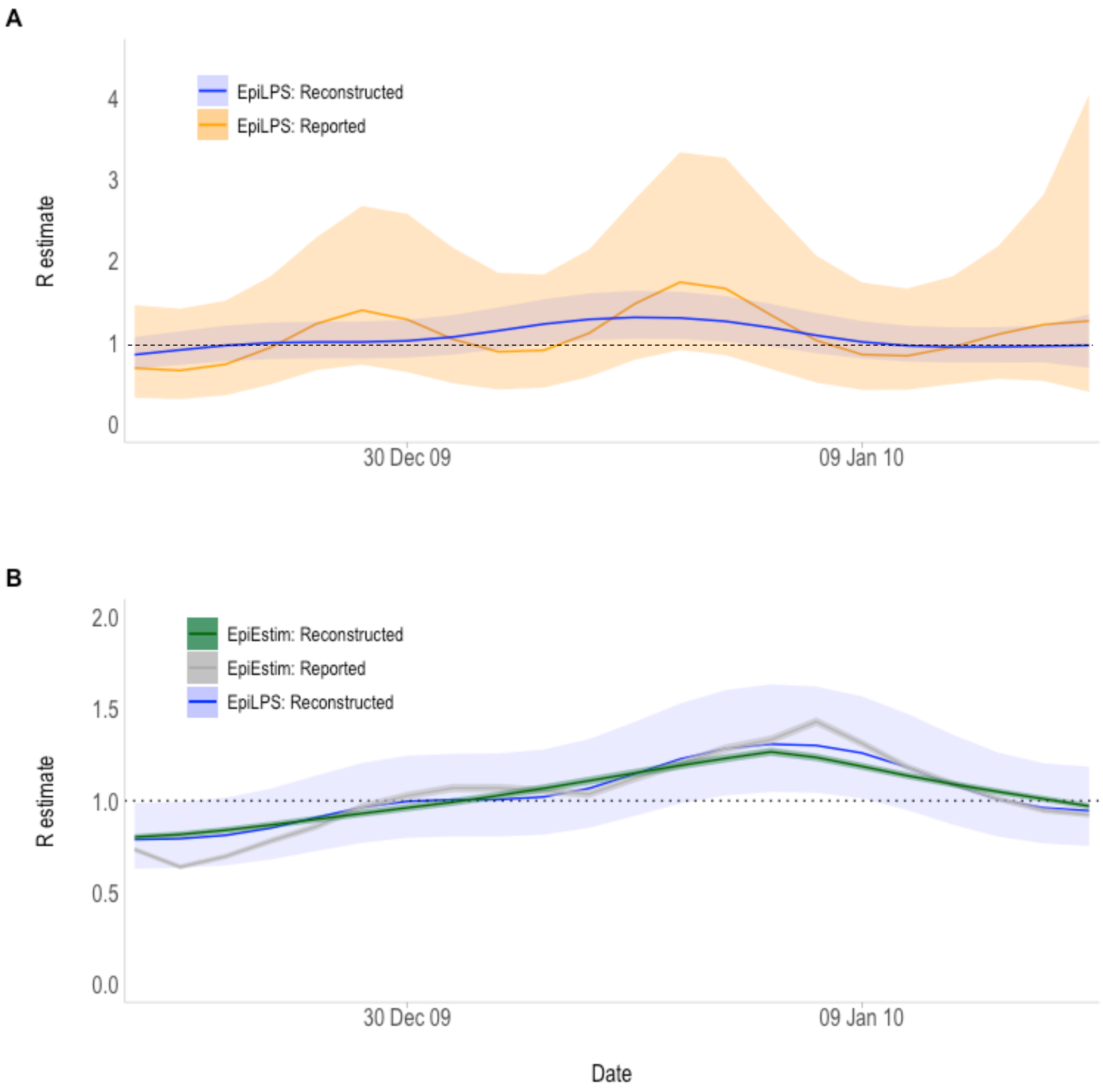

Figure S26. Supplying reconstructed daily influenza incidence data to EpiLPS. A) Comparison between default EpiLPS R_t estimates using reported daily incidence (orange) and daily incidence reconstructed using our EM algorithm (blue). B) Comparison between R_t estimates generated using EpiEstim with daily reported (grey) and daily reconstructed (green) incidence, with EpiLPS R_t estimates using daily incidence reconstructed using our EM algorithm (blue) shifted by 3 days. Lines represent mean R_t estimates, with the coloured shading representing 95% credible intervals.

References

1. Cori A, Ferguson NM, Fraser C, Cauchemez S. A New Framework and Software to Estimate Time-Varying Reproduction Numbers During Epidemics. Am J Epidemiol. 2013 Nov 1;178(9):1505–12.

2. Riley P, Cost AA, Riley S. Intra-Weekly Variations of Influenza-Like Illness in Military Populations. Mil Med. 2016 Apr 1;181(4):364–8.

3. Jombart T, Nouvellet P, Bhatia S, Kamvar ZN, Taylor T, Ghozzi S. projections: Project Future Case Incidence [Internet]. 2021 [cited 2022 Jun 9]. Available from: https://CRAN.R-project.org/package=projections

4. R Core Team. R: A language and environment for statistical computing. [Internet]. Vienna, Austria: R Foundation for Statistical Computing,; 2013. Available from: http://www.R-project.org/

5. Ferguson NM, Cucunubá ZM, Dorigatti I, Nedjati-Gilani GL, Donnelly CA, Basáñez MG, et al. Countering the zika epidemic in latin america. Science. 2016;353(6297):353–4.

6. Gressani O, Wallinga J, Althaus CL, Hens N, Faes C. EpiLPS: A fast and flexible Bayesian tool for estimation of the time-varying reproduction number. PLOS Comput Biol. 2022 Oct 10;18(10):e1010618.

List of Legends

Figure S1. The A) daily and B) weekly sliding R_t estimates for influenza based on the reported (grey) and the reconstructed (green) daily data by date of presentation at the military clinic. R_t estimates start on the first day of the second aggregation window (day 8 – 18th December 2009) and are plotted at the end of the time window. Shading corresponds to the 95% credible interval of the estimates.

Figure S2. A-B) The reported (grey) and reconstructed (green) daily incidence of influenza by the date of presentation at the military clinic, either on a natural scale (A) or log scale (B). C-D) The absolute difference in the C) daily and D) weekly sliding R_t estimates made using the reported daily data and the reconstructed daily data. Note that the y-axis scale is different in panels C and D.

Figure S3. The A) daily and B) weekly sliding R_t estimates for COVID-19 cases based on the reported (grey) and the reconstructed (green) daily data by date of specimen. R_t estimates start on the first day of the second aggregation window (day 8 – 28th February 2020) and are plotted at the end of the time window. Shading corresponds to the 95% credible interval of the estimates. The y-axis has been cropped to a maximum of 4 for clarity.

Figure S4. A-B) The reported (grey) and reconstructed (green) daily incidence of COVID-19 by the date of specimen, either on a natural scale (A) or log scale (B). C-D) The absolute difference in the C) daily and D) weekly sliding R_t estimates made using the reported data and the reconstructed data. Note that the y-axis scale is different in panels C and D.

Figure S5. The A) daily and B) weekly sliding R_t estimates for COVID-19 deaths based on the reported daily data (grey) and the reconstructed daily data (green) by date of death within 28 days of a positive test. R_t estimates start on the first day of the second aggregation window (day 8 – 9th March 2020) and are plotted at the end of the time window. Shading corresponds to the 95% credible interval of the estimates. The y-axis has been cropped to a maximum of 4 for clarity.

Figure S6. A-B) The reported (grey) and reconstructed (green) daily incidence of COVID-19 deaths within 28 days of a positive test, either on a natural scale (A) or log scale (B). C-D) The absolute difference in the C) daily and D) weekly sliding R_t estimates made using the reported data and the reconstructed data. Note that the y-axis scale is different in panels C and D.

Figure S7. The relative difference between the reported incidence for A) influenza, B) COVID-19 cases and C) COVID-19 deaths, on each weekday compared to the mean incidence for that week. Only data for full calendar weeks are included. Black lines represent the mean relative difference and the dashed grey line indicates zero (corresponding to no difference between the reported incidence on that day compared to the weekly mean).

Figure S8. Comparison of the percentage agreement in the classification of R_t estimates as above 1 (lower bound of the 95% CrI is above 1), encompassing 1, or below 1 (upper bound of the 95% CrI is below 1), when using reported and reconstructed incidence data. Daily and weekly sliding R_t estimates are compared for influenza cases (A-B), COVID-19 cases (C-D), and COVID-19 deaths (E-F). Due to low incidence, the first 30 days of data is excluded for both COVID-19 datasets. Darker shades of blue correspond to greater percentage agreement, with a strong correlation represented by a dark diagonal line across the grid squares from top left to bottom right.

Table S1. Time taken to estimate R_t in each of the real data scenarios.

Table S2. Definitions for the criteria used to assess the performance of our method in the simulation study.

Figure S9. Performance of the method when estimating a constant R_t for 100 simulated epidemics. A-D) The mean weekly sliding R_t estimates using daily data (grey) and reconstructed data (green). The black dashed line represents the true value of R_t that the 100 simulations are based on, which is either A) 1, B) 1.25, C) 1.5, or D) 1.75. The estimates and their 95% credible intervals (shaded areas) are very similar and therefore overlap. E-H) The bias (or absolute difference) in the mean R_t estimated from reconstructed data compared to the true value of R_t. The shaded area corresponds to the 95% quantiles

of the bias across the 100 simulations. I-L) The uncertainty (mean width of the 95% credible interval) in the R_t values estimated from reconstructed data. M-P) The proportion of the 100 R_t estimates obtained from reconstructed data where the 95% credible interval encompasses either the true value of R_t (black) or the value of R_t that would have been estimated from daily data (blue). Here, the 95% coverage is similar for both the true value of R_t and the daily data estimate, and therefore they overlap.

Figure S10. An example of reconstructed daily incidence data (on the log scale) with clear discontinuities on the borders between time periods where the data had been aggregated (grey dashed lines).

Figure S11. Performance of the method when estimating a time varying R_t for 100 simulated epidemics. In this scenario, R_t suddenly decreases on day 35 (grey dashed line) and remains constant before and after the step change. A-D) The mean weekly sliding R_t estimates using daily data (grey) and reconstructed data (green). The black dashed line represents the true value of R_t that the 100 simulations are based on, which is either a decrease from A) 1.25 to 0.75, B) 1.25 to 1, C) 1.5 to 1.25, or D) 1.75 to 1.5. The grey dotted line represents the threshold of $R_t = 1$. The estimates (plotted at the end of each time window) and their 95% credible intervals (shaded area) are very similar and therefore overlap. E-H) The bias (or absolute difference) in the mean R_t estimated from reconstructed data compared to the true value of R_t. The shaded area corresponds to the 95% quantiles of the bias across the 100 simulations. I-L) The uncertainty (mean width of the 95% credible interval) in the R_t values estimated from reconstructed data. M-P) The proportion of the 100 R_t estimates made using reconstructed data where the 95% credible interval encompasses either the true value of R_t (black) or the value of R_t that would have been estimated from daily data (blue).

Figure S12. Performance of the method when estimating a time varying R_t for 100 simulated epidemics. In this scenario, R_t suddenly increases on day 35 (grey dashed line) and remains constant before and after the step change. A-C) The mean weekly sliding R_t estimates using simulated daily data (grey) and reconstructed data (green). The black dashed line represents the true value of R_t that the 100 simulations are based on, which is either an increase from A) 1 to 1.25, B) 1.25 to 1.5, or C) 1.5 to 1.75. The grey dotted line represents the threshold of $R_t = 1$. The estimates (plotted at the end of each time window) and their 95% credible intervals (shaded area) are very similar and therefore overlap. D-F) The bias (or absolute difference) in the mean R_t estimated from reconstructed data compared to the true value of R_t. The shaded area corresponds to the 95% quantiles of the bias across the 100 simulations. G-I) The uncertainty (mean width of the 95% credible interval) in the R_t values estimated from reconstructed data. J-L) The proportion of the 100 R_t estimates made using reconstructed data where the 95% credible interval encompasses either the true value of R_t (black) or the value of R_t that would have been estimated from daily data (blue).

Figure S13. Performance of the method when estimating a time varying R_t for 100 simulated epidemics. In this scenario, R_t gradually decreases over the course of 30 days (day 20 to day 50, shown with grey dashed lines), and remains constant before and after the change. A-D) The mean weekly sliding R_t estimates using daily data (grey) and weekly data (green). The black dashed line represents the true value of R_t that the 100 simulations are based on, which is either a decline from A) 1.25 to 0.75, B) 1.25 to 1, C) 1.5 to 1.25, or D) 1.75 to 1.5. The grey dotted line represents the threshold of $R_t = 1$. The estimates (plotted at the end of each time window) and their 95% credible intervals (shaded area) are very similar and therefore overlap. E-H) The bias (or absolute difference) in the mean R_t estimated from weekly data compared to the true value of R_t. The shaded area corresponds to the 95% quantiles of the bias across the 100 simulations. I-L) The uncertainty (mean width of the 95% credible interval) in the R_t values estimated from weekly data. M-P) The proportion of the 100 R_t estimates made using weekly data where the 95% credible interval encompasses either the true value of R_t (black) or the value of R_t that would have been estimated from daily data (blue).

Figure S14. Performance of the method when estimating a time varying R_t for 100 simulated epidemics. In this scenario, R_t gradually increases over the course of 30 days (day 20 to day 50, shown with grey dashed lines), and remains constant before and after the change. A-C) The mean weekly sliding R_t estimates using daily data (grey) and weekly data (green). The black dashed line represents the true value of R_t that the 100 simulations are based on, which is either an increase from A) 1 to 1.25, B) 1.25 to 1.5, or C) 1.5 to 1.75. The grey dotted line represents the threshold of $R_t = 1$. The estimates (plotted in the middle of each time window) and their 95% credible intervals (shaded area) are very similar and therefore overlap. D-F) The bias (or absolute difference) in the mean R_t estimated from weekly data compared to the true value of R_t. The

shaded area corresponds to the 95% quantiles of the bias across the 100 simulations. G-I) The uncertainty (mean width of the 95% credible interval) in the R_t values estimated from weekly data. J-L) The proportion of the 100 R_t estimates made using weekly data where the 95% credible interval encompasses either the true value of R_t (black) or the value of R_t that would have been estimated from daily data (blue).

Figure S15. Plotting the gradual change in R_t scenarios for 100 simulated epidemics in the middle of the time window. Here, the gradually increasing (A-C) and decreasing (G-J) R_t estimate plots are exactly as in figures S13 and S14, except they are plotted at t-3.5. Each 95% coverage plot (D-F & K-N) corresponds to the plot directly above and shows the proportion of the 100 R_t estimates made using weekly data where the 95% credible interval encompasses either the true value of R_t (black) or the value of R_t that would have been estimated from daily data (blue). As a reference, the 95% coverage of the true value of R_t for the R_t estimates plotted at the end of the time window (see figures S13 & S14) are shown in grey.

Figure S16. Plotting the stepwise change in R_t scenarios for 100 simulated epidemics in the middle of the time window. Here, the stepwise increasing (A-C) and decreasing (G-J) R_t estimate plots are exactly as in figures S11 and S12, except they are plotted at t-3.5. Each 95% coverage plot (D-F & K-N) corresponds to the plot directly above and shows the proportion of the 100 R_t estimates made using weekly data where the 95% credible interval encompasses either the true value of R_t (black) or the value of R_t that would have been estimated from daily data (blue). As a reference, the 95% coverage of the true value of R_t for the R_t estimates plotted at the end of the time window (see figures S11 & S12) are shown in grey.

Figure S17. Assessing the performance of our method in the presence of weekend effects in the reported data. A-C) Example of reported (grey) and reconstructed (green) daily incidence data taken from one of the 100 simulated epidemics (selected at random) for a (A) constant R_t of 1.5, (B) stepwise decrease in R_t from 1.5 to 1.25, and (C) gradual increase in R_t from 1.25 to 1.5. R_t was estimated over D-F) daily, G-I) weekly, and J-L) two-weekly sliding time windows. The black dashed line is the true value of R_t that the simulated data was based on. Note: y-axis limits for each incidence plot varies and the y-axis scale is different for the daily R_t estimate plots (second row).

Figure S18. Assessing the ability to detect genuine mid-aggregation window variations in transmissibility (either increasing (left), or decreasing (right) on weekends) from reconstructed daily data (green) and the true simulated daily data (grey), with R_t estimated over daily (A-B) or weekly-sliding (C-D) time windows.

Figure S19. The mean weekly sliding R_t estimates generated after each of the 10 iterations from 100 stochastically simulated epidemics for the following scenarios: A) constant R_t of 1.5, B) stepwise decrease in R_t from 1.5 to 1.25, and C) gradual increase in R_t from 1.25 to 1.5.

Figure S20. Performance of the method using alternative aggregations of incidence data when the end of aggregation windows <u>align</u> with the step change on day 35. We consider the following scenarios: a constant R_t of 1.5 (left), stepwise decrease in R_t from 1.5 to 1.25 (middle), and a gradual increase in R_t from 1.25 to 1.5 (right), with data aggregated over A-C) 3 days, D-F) 7 days, G-I) 10 days, and J-L) 14 days. For the 10-day and 14-day aggregations of data, we compare estimates made using weekly sliding windows (green) with sliding windows of length matching that of the aggregation of data (blue). For all scenarios, R_t estimates are plotted at the end of the sliding time window. Grey dotted lines show the end of each aggregation window and the grey shaded areas are the time periods excluded in order to align the aggregation windows (if necessary).

Figure S21. Performance of the method using alternative aggregations of incidence data when the end of aggregation windows <u>do not align</u> with the step change on day 35. We consider the following scenarios: a constant R_t of 1.5 (left), stepwise decrease in R_t from 1.5 to 1.25 (middle), and a gradual increase in R_t from 1.25 to 1.5 (right), with data aggregated over A-C) 3-days, D-F) 7-days, G-I) 10-days, and J-L) 14-days. For the 10-day and 14-day aggregations of data, we compare estimates made using weekly sliding windows (green) with sliding windows of length matching that of the aggregation of data (blue). For all scenarios, R_t estimates are plotted at the end of the sliding time window. Grey dotted

lines show the end of each aggregation window and the grey shaded areas are the time periods excluded in order to misalign the aggregation windows (if necessary).

Figure S22. Comparison between the reconstructed daily incidence generated by the EM algorithm and LOESS smoothing, based on smoothing points (black circles) at either the end (A – days 7, 14, 21, 28 and 35) or middle (B - days 3, 10, 17, 24, 31) of aggregation time windows. The "observed" incidence at the smoothing points was computed as the naïve disaggregation of the weekly incidence for that week. The reported daily incidence of influenza (grey), the daily reconstructed incidence generated by the EM algorithm (black), and the daily reconstructed incidence generated by LOESS smoothing with the degree of the polynomial set to either 0 (red), 1 (blue), or 2 (magenta). Note that LOESS smoothing does not allow interpolation beyond the observations.

Table S3. Differences between the re-aggregated reconstructed daily incidence data generated by either the EM algorithm method or LOESS smoothing. "True weekly" is the weekly aggregations of the reported daily influenza dataset that would be supplied by the user.

Figure S23. Comparison between R_t estimates made using reconstructed daily incidence generated by the EM algorithm (black), or by LOESS smoothing with the degree of the polynomial set to either 0 (red), 1 (blue), or 2 (magenta). The LOESS approach is based on smoothing points at the end (A) or middle (B) of aggregation time windows.

Figure S24. The absolute difference between R_t estimates made using the reported daily data and the reconstructed daily incidence, generated by the EM algorithm (black), or by LOESS smoothing with the degree of the polynomial set to either 0 (red), 1 (blue), or 2 (magenta). The grey dashed line represents an absolute difference of 0. The LOESS approach is based on smoothing points at the end (A) or middle (B) of aggregation time windows.

Figure S25. Comparison of R_t estimates obtained using a workaround approach (where a weekly SI is supplied to match the incidence data) and the EM approach. For clarity, we show the R_t estimates between 19th December 2015 and 15th April 2016, when R_t is estimated to have fallen below 4.

Figure S26. Supplying reconstructed daily influenza incidence data to EpiLPS. A) Comparison between default EpiLPS R_t estimates using reported daily incidence (orange) and daily incidence reconstructed using our EM algorithm (blue). B) Comparison between R_t estimates generated using EpiEstim with daily reported (grey) and daily reconstructed (green) incidence, with EpiLPS R_t estimates using daily incidence reconstructed using our EM algorithm (blue) shifted by 3 days. Lines represent mean R_t estimates, with the coloured shading representing 95% credible intervals.
